# Analysis of pain intensity and quality of life in patients with lumbar disc disease treated with PLIF (Posterior Lumbar Interbody Fusion) and ALIF (Anterior Lumbar Interbody Fusion) interbody stabilization methods: a 3-month retrospective analysis

**DOI:** 10.7717/peerj.21582

**Published:** 2026-08-06

**Authors:** Kazimiera Hebel, Sylwia Jałtuszewska, Monika Dydyna, Katarzyna Kubała, Aleksandra Bryndal

**Affiliations:** 1 Institute of Health Sciences, Pomeranian University of Slupsk, Slupsk, Poland; 2 The Faculty of Health Sciences with the Institute of Maritime and Tropical Medicine, Medical University of Gdansk, Gdansk, Poland; 3 Provincial Specialist Hospital named after Janusz Korczak in Slupsk, Slupsk, Poland; 4 State Higher School of Vocational Education in Koszalin, Koszalin, Poland

**Keywords:** Lumbar disc disease, Interbody stabilization, PLIF, ALIF, Spinal pain, Quality of life

## Abstract

**Background:**

Lumbar disc disease is one of the most common causes of spinal pain and represents a significant health problem. Surgical treatment often involves interbody stabilization, performed using Posterior Lumbar Interbody Fusion (PLIF) or Anterior Lumbar Interbody Fusion (ALIF) methods.

**Methods:**

A retrospective cohort study was conducted on 112 patients (64 women, 48 men) with lumbar disc disease aged 17–72 years. Patients underwent PLIF (73 patients, 65.18%) or ALIF (39 patients, 34.82%) procedures. Pain assessment utilized the visual analogue scale (VAS) scale and numerical rating scale (NRS) for specific disc disease symptoms. Quality of life was measured using Cantril’s ladder retrospectively before symptom onset, 3 months post-operation, and prospectively for predicted future (2–3 years). Robust linear regression models with interaction analysis were applied.

**Results:**

Significant pain reduction on the VAS scale was found after 3 months (average 6.07 points, *p* < 0.001). Both procedures demonstrated significant pain reduction at 3 months. Pain outcomes varied by symptom type, with substantial improvements observed in overall pain, spinal pain, limb pain, and numbness across both surgical approaches. Most symptoms showed very high effect sizes (r ≥ 0.97), except for spinal stiffness. Quality of life significantly improved over time (*p* < 0.001), with higher baseline values after ALIF (0.66 points, *p* = 0.001). Physical activity improved quality of life, while tobacco smoking increased spinal stiffness.

**Conclusions:**

Both PLIF and ALIF demonstrated substantial reductions in pain complaints and improvements in quality of life in patients with lumbar disc disease in 3-month follow-up. Both PLIF and ALIF demonstrated substantial pain reduction and quality of life improvement in 3-month follow-up. However, definitive recommendations for method selection require long-term studies with patient randomization and longer follow-up periods.

## Introduction

Lumbar disc disease represents one of the most common causes of chronic spinal pain and significantly impacts the functioning and quality of life of patients worldwide. Globally, in 2020, nearly 10% of the world’s population suffered from lower back pain, and projections indicate that this number will rise to 36.4% by 2050 (GBD 2021 Low Back Pain Collaborators, 2023).

In Poland, complaints related to lumbosacral spine pain affect 38.4% of women and 21.2% of men (Meucci, Fassa & Faria, 2015; Raciborski, Gasik & Kłak, 2016). Chronic and recurrent back pain can affect up to 60% of the population and is the most common reason for seeking primary care physicians (Citko et al., 2017). This disease has been classified as a lifestyle disease, and its development is favored by factors specific to modern lifestyle such as forced body position, lack of physical activity, stress, and improper diet (GBD 2021 Low Back Pain Collaborators, 2023).

The pathological process of the intervertebral disc presents a complex degenerative picture that begins as early as adolescence. Studies indicate that 20% of young people already have mild symptoms of the disease, which progresses with age (Farag et al., 2024; Kos, Gradisnik & Velnar, 2019). Intervertebral discs, in addition to connecting vertebral bodies, perform a shock-absorbing function. Excessive loads lead to destruction of the annulus fibrosus and displacement of the nucleus pulposus, which consequently causes compression of nerve structures and triggers pain (Yurube et al., 2023).

Treatment of disc disease includes pharmacotherapy, rehabilitation, and in cases of conservative treatment failure, surgical intervention. One method of surgical treatment is interbody stabilization, which involves removal of the degenerated fragment or entire disc and implantation of an interbody implant. This procedure aims to reduce compression on nerve structures, prevent disc re-herniation, and stabilize the spine (Mosaad et al., 2023; Lee et al., 2024).

While discectomy remains a common approach for acute disc herniations, evidence suggests primary fusion may provide superior outcomes in patients with significant degeneration, instability, or recurrent herniation (Phan et al., 2017; Jannelli et al., 2025; Mobbs et al., 2015). The selection between discectomy and fusion must be individualized based on degenerative severity, segmental stability, and previous surgical history.

Interbody stabilization can be performed using two main methods: Posterior Lumbar Interbody Fusion (PLIF)—stabilization through posterior approach, and Anterior Lumbar Interbody Fusion (ALIF)—stabilization through anterior approach.

ALIF offers several advantages including the ability to place larger interbody implants which translates to better load distribution and fusion surface area (Scott-Young et al., 2024; Odeh et al., 2020), preservation of posterior paraspinal muscles and ligamentous structures (Veliky et al., 2024), and more effective indirect decompression through restoration of intervertebral disc height leading to widening of intervertebral foramina (Odeh et al., 2020; Tung et al., 2023). However, ALIF requires specialized anterior surgical approach with potential risks including vascular complications and retrograde ejaculation in males (Mobbs et al., 2015).

PLIF provides direct access for neural decompression through a familiar posterior approach, allows simultaneous treatment of spinal canal stenosis, and enables bilateral cage placement (Mobbs et al., 2015; Rathbone et al., 2023). The main limitations of PLIF include disruption of posterior muscular and ligamentous structures, increased epidural scarring, potential neural retraction risks, and placement of smaller interbody devices due to anatomical constraints imposed by the neural elements (Rathbone et al., 2023; Veliky et al., 2024).

The choice of appropriate technique depends on many factors, including the patient’s clinical condition, location and characteristics of pathology, presence of spinal canal stenosis requiring posterior decompression, and the surgeon’s experience and preferences (Mobbs et al., 2015; Rathbone et al., 2023; Young et al., 2008).

Surgical treatment of lumbar disc disease using interbody stabilization methods remains a significant therapeutic challenge, requiring in-depth analysis of clinical outcomes in patients. Despite the widespread use of both techniques, there is limited evidence comparing their clinical outcomes in routine clinical practice, particularly regarding pain reduction patterns and quality of life improvement in short-term follow-up. The aim of this study was to analyze the intensity of pain complaints and evaluate life satisfaction in patients with lumbar disc disease after surgical treatment using interbody stabilization methods PLIF and ALIF, taking into account demographic and clinical factors affecting these parameters in a 3-month follow-up period. Based on existing literature suggesting biomechanical and anatomical advantages of ALIF—including the capacity for larger implant placement, preservation of posterior spinal structures, and more effective restoration of disc height and foraminal volume—we hypothesized that ALIF would demonstrate greater pain reduction and quality of life improvement compared to PLIF in the 3-month postoperative period. We further hypothesized that both surgical approaches would result in clinically significant improvement from baseline, with patient-specific factors such as age, body mass index (BMI), and lifestyle potentially influencing the magnitude of treatment response.

## Materials and Methods

A retrospective observational cohort study was conducted to evaluate clinical outcomes in 112 patients with lumbar disc disease who underwent interbody stabilization procedures using PLIF or ALIF methods. The study included patients treated between 2021–2022 at the Regional Specialist Hospital in Słupsk in Poland.

### Inclusion and exclusion criteria

Inclusion criteria were: (1) lumbar disc disease with significant degeneration (Pfirrmann grade ≥3), segmental instability, recurrent herniation, or chronic discogenic pain >6 months; (2) age ≥17 years; (3) failed conservative treatment (minimum 3 months); (4) qualification for PLIF or ALIF based on institutional protocols; and (5) complete documentation. The decision for fusion over simple discectomy reflected protocols recognizing limited long-term effectiveness of discectomy in patients with advanced degeneration or instability (Karadağ et al., 2022). Exclusion criteria were: unavailability of data at required time points, incomplete documentation, other concurrent spinal procedures.

### Research methods

To assess pain intensity before surgery and 3 months postoperatively, a 10-point visual analogue scale (VAS) (Delgado et al., 2018) and an 11-point numerical rating scale (NRS) for specific disc disease symptoms were used, including: sciatica with radiation to the lower limb, lumbosacral spine pain, lower limb pain (independent of sciatica), numbness and paresthesias in lower limbs, impairment of superficial and deep sensation, motor deficits in lower limb muscles, stiffness and limitation of spinal mobility, foot drop (Bielewicz, Daniluk & Kamieniak, 2022). Each symptom was assessed independently using separate NRS scales, allowing detailed evaluation of individual symptom trajectories.

Life satisfaction was measured using Cantril’s ladder at three time points: retrospectively: before symptom onset, 3 months postoperatively, and prospectively: predicted for the future (2–3 years after treatment). Cantril’s ladder has a graphical form of a ladder with steps marked with consecutive numbers from 0 to 10. Next to the ladder is explanatory text stating that number 10 means the best possible life, while number 0 means the worst life. Respondents’ task was to evaluate their current life and place an “X” mark in the appropriate place on the ladder. It is accepted that results below 6 indicate life dissatisfaction, while values ≥6 are interpreted as life satisfaction according to validation by Mazur et al. (2018). Instructions for patients were standardized in all cases.

All data were retrospectively collected from hospital medical records, preoperative assessment sheets, surgical procedure protocols, postoperative follow-up documentation, and patient self-assessment questionnaires. The choice of a 3-month observation period was dictated by several factors. First, literature indicates that the most intensive changes in neurological and pain symptom severity after interbody stabilization occur in the first 3–6 months after surgery (Anzai et al., 2024). Second, analysis of available comparative studies PLIF *vs*. ALIF shows that early results (3–6 months) strongly correlate with long-term outcomes (Rathbone et al., 2023). Third, limitations of the retrospective study design required choosing a time point with the greatest availability of complete medical documentation.

The choice of surgical method (PLIF *vs*. ALIF) was made by the neurosurgical team based on the following factors. ALIF was preferentially used for discogenic low back pain with coexisting radicular pain, degenerative disease of 1–2 adjacent intervertebral discs (mainly L4-L5, L5-S1), absence of spinal canal pathology requiring posterior approach, non-obese patients, and possibility of safe retroperitoneal approach. PLIF was used for spinal canal stenosis, nucleus pulposus herniation with compression of posterior structures, lumbar instability, contraindications to anterior approach, and need for simultaneous posterior decompression. All procedures were performed by experienced neurosurgeons (>50 cases/year). Standard PLIF involved bilateral laminectomy, facetectomy, discectomy, interbody cage placement, and posterior instrumentation. ALIF involved retroperitoneal approach, anterior discectomy, cage placement, with or without posterior supplemental fixation. Postoperative rehabilitation included early mobilization (day 1–2), physical therapy initiation at 4–6 weeks, and gradual return to activities over 3 months under physiotherapist supervision. This non-randomized selection based on clinical judgment introduces potential bias but reflects real-world surgical practice and our institutional preference for fusion in appropriately selected patients.

The study was conducted in accordance with the principles of the Declaration of Helsinki. The research protocol was approved by the University Ethics Committee for Scientific Research at Pomeranian University in Słupsk—UKEBN/3/2024. Due to the retrospective nature of medical record analysis, additional patient consent for study participation was not required.

### Statistical analysis

The level of statistical significance was set at α = 0.05. Normality of distribution of numerical variables was assessed using the Shapiro-Wilk test. Data with normal distribution were presented as mean ± standard deviation, while data with non-normal distribution as median and interquartile ranges (Q1, Q3). Categorical variables were presented as frequency (percentage). The Wilcoxon rank test was used to compare two independent groups with non-parametric distribution. When assessing intragroup differences at two time points, the Wilcoxon test for paired observations was used with calculation of the two-point rank correlation coefficient as a measure of effect size. For analysis of intragroup differences at three time points (before symptom onset, 3 months post-surgery, and future prognosis), the Friedman test with Durbin-Conover *post-hoc* tests was used. *p*-values were corrected using the Holm method to control for multiple comparison error. Effect size was assessed using Kendall’s W coefficient. To estimate the influence of multiple factors on outcome variables, robust linear regression (robust linear modeling, RLM) with M-estimator through iteratively reweighted least squares (IWLS) was used. This method was chosen due to its robustness to outliers and lack of requirement for normality of residual distribution. The models included the following predictors: age (centered relative to median), sex, BMI, tobacco smoking, physical activity, type of procedure (PLIF *vs*. ALIF), and measurement time point. Interactions between time point and procedure type were analyzed. Results were presented as unstandardized regression coefficients (B) with 95% confidence intervals. Analyses were performed in R environment version 4.3.1 (R Core Team, 2023) using packages: MASS (robust regression), emmeans (*post-hoc* comparisons), gtsummary (descriptive tables), ggstatsplot (visualizations), and dplyr (data manipulation). All analyses were performed at significance level *p* < 0.05.

## Results

### Study group characteristics

The study included 112 patients with lumbar disc disease (64 women, 57.1%; and 48 men, 42.9%) aged from 17 to 72 years (median 43 years). Patients underwent interbody stabilization procedures using the PLIF method (73 patients, 65.18%) or ALIF method (39 patients, 34.82%). Statistically significant differences were observed between sexes in the choice of surgical method (*p* < 0.001). The ALIF procedure was significantly more frequently used in women (84.6%) than in men (15.4%), while PLIF was more frequently used in men (57.5%) than in women (42.5%).

Among the study participants, overweight individuals (43 people; 38.39%) and those with normal body weight (39 people, 34.82%) predominated, and grade I obesity was found in 28 people (25.00%). Nicotine addiction was declared by 55 people (49.11%). Comorbidities included: hypothyroidism (18 people, 16.07%), arterial hypertension (14 people, 12.50%), and diabetes (3 people, 2.68%). Professional activity was declared by 83 patients (74.11%), of whom 69 people (61.61%) performed physical work, eight people performed office work (7.14%), and six people (5.36%) performed other types of work. A detailed demographic characterization of the study group is presented in Table 1.

**Table 1:** Demographic characteristics, lifestyle, and comorbidities of the entire sample and by sex, ALIF and PLIF.

Characteristic	Total (*n* = 112)	Women (*n* = 64)	Men (*n* = 48)	*p*	ALIF (*n* = 39)	PLIF (*n* = 73)	*p*
**Demographics**							
Age (years)^b^	43.0 (36.0–55.0)	45.0 (36.8–56.0)	43.0 (35.0–52.0)	0.497^d^	43.0 (38.5–50.5)	43.0 (35.0–56.0)	0.723^d^
Age categories^a^:				0.601			0.064
≤30 years	16 (14.3%)	7 (10.9%)	9 (18.8%)		3 (7.7%)	13 (17.8%)	
31–45 years	48 (42.9%)	27 (42.2%)	21 (43.8%)		20 (51.3%)	28 (38.4%)	
46–60 years	36 (32.1%)	23 (35.9%)	13 (27.1%)		15 (38.5%)	21 (28.8%)	
≥61 years	12 (10.7%)	7 (10.9%)	5 (10.4%)		1 (2.6%)	11 (15.1%)	
Sex:^a^				–			<0.001^c^
Female	64 (57.1%)	64 (57.1%)	–		33 (84.6%)	31 (42.5%)	
Male	48 (42.9%)	–	48 (42.9%)		6 (15.4%)	42 (57.5%)	
BMI, kg/m²^b^	27.0 (23.0–30.0)	26.0 (23.0–29.3)	27.0 (24.0–31.0)	0.394^d^	25.0 (23.0–28.5)	27.0 (24.0–31.0)	0.086^d^
BMI categories^a^:				0.216			0.245
Normal	39 (34.8%)	26 (40.6%)	13 (27.1%)		18 (46.2%)	21 (28.8%)	
Overweight	43 (38.4%)	23 (35.9%)	20 (41.7%)		13 (33.3%)	30 (41.1%)	
Obesity I°	28 (25.0%)	13 (20.3%)	15 (31.3%)		8 (20.5%)	20 (27.4%)	
Obesity II°	2 (1.8%)	2 (3.1%)	0 (0.0%)		0 (0.0%)	2 (2.7%)	
**Lifestyle^a^**							
Smoking	55 (49.1%)	31 (48.4%)	24 (50.0%)	0.870	20 (51.3%)	35 (47.9%)	0.843
**Comorbidities^a^**							
Hypothyroidism	18 (16.1%)	13 (20.3%)	5 (10.4%)	0.158	8 (20.5%)	10 (13.7%)	0.506^c^
Arterial hypertension	14 (12.5%)	10 (15.6%)	4 (8.3%)	0.248	6 (15.4%)	8 (11.0%)	0.708^c^
Diabetes	3 (2.7%)	1 (1.6%)	2 (4.2%)	0.575^c^	1 (2.6%)	2 (2.7%)	1.000^c^
**Work^a^**							
Professional activity	83 (74.1%)	45 (70.3%)	38 (79.2%)	0.290	30 (76.9%)	53 (72.6%)	0.786
Physical work	69 (61.6%)	37 (57.8%)	32 (66.7%)	0.340	24 (61.5%)	45 (61.6%)	0.361

**Notes:**

a n (%).

b Mdn (Q1, Q3).

c Fisher’s exact test.

d Wilcoxon rank test.

Baseline comparison between surgical groups (Table 1) revealed that despite significant sex distribution differences (*p* < 0.001), ALIF and PLIF groups were comparable in age (median 43 years in both groups), BMI (ALIF: 25.0 kg/m², PLIF: 27.0 kg/m²), smoking prevalence (ALIF: 51.3%, PLIF: 47.9%), comorbidities including hypothyroidism, arterial hypertension, and diabetes, professional activity, and physical work. This baseline comparability in most demographic and clinical characteristics, with the exception of sex distribution, provides a reasonable foundation for outcome comparison while acknowledging the inherent selection bias in surgical approach assignment.

### Preoperative neurological assessment

Analysis of neurological symptoms before surgery showed that motor disorders occurred in nine patients (8.04%), sensory disorders in 14 patients (12.50%), and limb numbness in 72 patients (64.29%). Spinal pain was reported by 98 patients (87.50%), lower limb pain by 85 patients (75.89%), and sphincter dysfunction by five patients (4.46%). No significant differences were found in the frequency of neurological symptoms between patients qualified for PLIF and ALIF procedures (all *p* > 0.05).

### Multifactorial analysis of pain intensity—VAS scale

Regression analysis demonstrated significant pain reduction after surgical intervention (Table 2). Baseline pain intensity was 8.07 points (95% confidence interval (CI) [7.56–8.58], *p* < 0.001). Multifactorial analysis showed significant pain reduction at 3 months by 6.07 points (95% CI [−6.67 to −5.48], *p* < 0.001), with no significant effect of sex (B = –0.15; 95% CI [−0.54 to 0.23], *p* = 0.434), age (B = 0.01; 95% CI [−0.00 to 0.03], *p* = 0.103), tobacco smoking (B = 0.18; 95% CI [−0.17 to 0.53], *p* = 0.313), or physical work (B = 0.03; 95% CI [−0.34 to 0.39], *p* = 0.882) on pain intensity.

**Table 2:** Multifactorial robust regression analysis for pain intensity on the VAS scale.

Predictor	B	95% CI	*p*
Constant (intercept)	8.07	[7.56–8.58]	<0.001
**Time**			
Baseline	ref	–	–
3 months post-surgery	−6.07	[−6.67 to −5.48]	<0.001
**Sex**			
Female	ref	–	–
Male	−0.15	[−0.54 to 0.23]	0.434
**Age** (centered, Mdn = 43 years)	0.01	[−0.00 to 0.03]	0.103
**BMI** (centered)	−0.01	[−0.04 to 0.02]	0.606
**Tobacco smoking** (yes *vs*. no)	0.18	[−0.17 to 0.53]	0.313
**Physical work** (yes *vs*. no)	0.03	[−0.34 to 0.39]	0.882
**Type of procedure**			
ALIF	ref	–	–
PLIF	−0.11	[−0.66 to 0.44]	0.690
**Interaction**			
3 months × PLIF	1.66	[0.93–2.40]	<0.001
**Model fit: R^2^ = 0.80**			

**Notes:**

N_obs = 224 (112 patients × Two time points).

B—unstandardized regression coefficient; CI—confidence interval; ref—reference level. Model: robust linear regression (RLM) with M-estimator.

**Figure 1:**
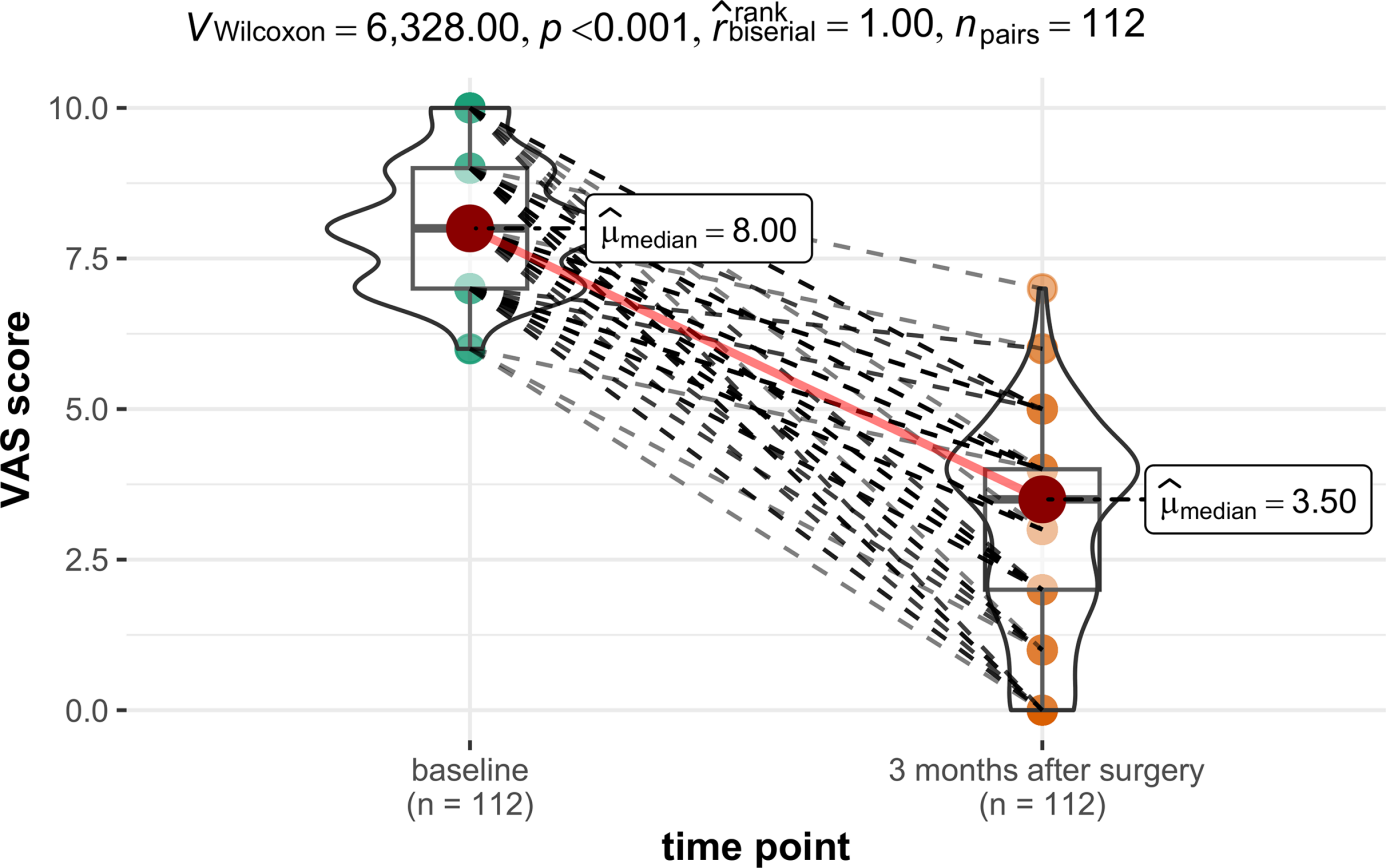
Temporal dynamics of VAS scores in discopathy patients.

Patients after PLIF showed less pain reduction compared to ALIF (B = 1.66; 95% CI [0.93–2.40], *p* < 0.001). This means that while both procedures demonstrated substantial pain reduction, ALIF was associated with greater pain reduction than PLIF. The temporal dynamics of pain intensity on the VAS scale with comparison of both surgical methods is presented in Fig. 1.

### Analysis of specific disc disease symptoms

Detailed regression analysis for individual disc disease symptoms revealed different patterns of response to surgical treatment. In the assessment of sciatica, baseline intensity was 7.52 points (95% CI [6.86–8.19], *p* < 0.001) with significant reduction at 3 months by 7.13 points (95% CI [−7.90 to −6.36], *p* < 0.001). Interestingly, it was found that each year of age reduced sciatica intensity by 0.02 points (95% CI [−0.04 to −0.00], *p* = 0.019), while sex analysis showed no significant effect.

Spinal pain was characterized by baseline intensity of 8.29 points (95% CI [7.66–8.92], *p* < 0.001) with reduction at 3 months by 5.18 points (95% CI [−5.92 to −4.45], *p* < 0.001). In contrast to sciatica, older patients experienced greater spinal pain (B = 0.02; 95% CI [0.00 to 0.04], *p* = 0.015). Similar to the overall VAS scale, an interaction was found indicating less spinal pain reduction after PLIF compared to ALIF (B = 1.44; 95% CI [0.53 to 2.34], *p* = 0.002).

Lower limb pain showed significant reduction after surgery by 7.78 points (95% CI [−8.49 to −7.07], *p* < 0.001). Patients after PLIF had lower baseline limb pain intensity compared to ALIF (B = −0.93; 95% CI [−1.59 to −0.27], *p* = 0.006), but also less reduction over time (B = 1.41; 95% CI [0.53–2.29], *p* = 0.002).

Limb numbness was characterized by baseline intensity of 4.15 points (95% CI [3.23–5.08], *p* < 0.001) with reduction at 3 months by 3.77 points (95% CI [−4.85 to −2.70], *p* < 0.001). Unlike other symptoms, patients after PLIF had higher baseline numbness (B = 1.98; 95% CI [0.98–2.97], *p* < 0.001), but also greater improvement over time (B = −1.45; 95% CI [−2.78 to −0.12], *p* = 0.032).

Spinal stiffness was an exception as the only symptom that did not show significant improvement at 3 months (B = −0.56; 95% CI [−1.91 to 0.79], *p* = 0.414). Significant factors affecting stiffness included age, which increased stiffness (B = 0.05; 95% CI [0.02–0.09], *p* = 0.002), BMI, which decreased stiffness (B = −0.10; 95% CI [−0.19 to −0.01], *p* = 0.027), and tobacco smoking, which significantly increased stiffness (B = 1.53; 95% CI [0.73–2.33], *p* < 0.001).

### Quality of life analysis

Multifactorial regression analysis of quality of life included 336 observations (112 patients × 3 time points) and revealed complex patterns of change (Table 3). Baseline quality of life level was 7.44 points (95% CI [6.43–8.45], *p* < 0.001).

**Table 3:** Multifactorial robust regression analysis for quality of life (Cantril’s ladder).

Predictor	B	95% CI	*p*
**Constant (intercept)**	**7.44**	**[6.43–8.45]**	**<0.001**
**Time point **			
Before symptom onset	ref	—	—
3 months post-surgery	0.25	[−0.16 to 0.67]	0.236
Future prognosis	**0.68**	**[0.27–1.10]**	**0.001**
**Demographic factors**			
**Sex** (male *vs*. female)	0.06	[−0.17 to 0.29]	0.613
**Age** (centered, Mdn = 43 years)	**−0.01**	**[−0.02 to −0.00]**	**0.003**
**BMI** (centered, Mdn = 27.0 kg/m²)	0.00	[−0.01 to 0.02]	0.747
**Behavioral factors**			
**Smoking** (yes *vs*. no)	0.14	[−0.07 to 0.34]	0.188
**Physical activity** (yes *vs*. no)	**0.33**	**[0.10–0.57]**	**0.006**
**Physical work** (yes *vs*. no)	−0.11	[−0.36 to 0.14]	0.389
**Type of procedure**			
**PLIF *vs*. ALIF**	**−0.66**	**[−1.04 to −0.28]**	**0.001**
**Neurological symptoms**			
**Motor disorders** (yes *vs*. no)	**−0.43**	**[−0.83 to −0.03]**	**0.036**
**Limb numbness** (yes *vs*. no)	**−0.31**	**[−0.56 to −0.05]**	**0.018**
**Spinal pain** (VAS intensity)	**−0.64**	**[−0.97 to −0.31]**	**<0.001**
**Relationship of baseline pain with quality of life**			
**Baseline VAS pain**	**0.20**	**[0.10–0.31]**	**<0.001**
**Model fit: R^2^ = 0.31**			

**Notes:**

N_obs= 336 (112 patients × three time points); B—unstandardized regression coefficient; ref—reference level; Model: robust linear regression (RLM) with M-estimator. Bold values indicate statistically significant results (*p* < 0.05).

Analysis of temporal dynamics showed no significant change in quality of life 3 months after surgery (B = 0.25; 95% CI [−0.16 to 0.67], *p* = 0.236), while future prognosis indicated significant improvement (B = 0.68; 95% CI [0.27–1.10], *p* = 0.001).

Among demographic and behavioral factors, each year of age decreased quality of life by 0.01 points (95% CI [−0.02 to −0.00], *p* = 0.003), while sex and BMI showed no significant effect. Tobacco smoking also did not significantly affect quality of life (B = 0.14; 95% CI [−0.07 to 0.34], *p* = 0.188), while physical activity significantly improved it (B = 0.33; 95% CI [0.10–0.57], *p* = 0.006).

Type of surgical procedure showed significant effect on quality of life, with patients after PLIF reporting lower baseline quality of life compared to ALIF (B = −0.66; 95% CI [−1.04 to −0.28], *p* = 0.001). Additionally, motor disorders decreased quality of life (B = −0.43; 95% CI [−0.83 to −0.03], *p* = 0.036), as did spinal pain (B = −0.64; 95% CI [−0.97 to −0.31], *p* < 0.001).

A positive relationship was also observed between baseline pain intensity on the VAS scale and quality of life (B = 0.20; 95% CI [0.10–0.31], *p* < 0.001).

### Temporal dynamics of symptoms and life satisfaction

Analysis of paired comparisons before surgery and at 3 months showed significant improvements in most disc disease symptoms. Median pain intensity on the VAS scale decreased from 8.00 to 3.50 points (*p* < 0.001), sciatica from 8.00 to 0.00 points (*p* < 0.001), spinal pain from 8.00 to 4.00 points (*p* < 0.001), and limb pain from 8.00 to 0.00 points (*p* < 0.001). Limb numbness decreased from 7.00 to 0.00 points (*p* < 0.001). All main symptoms showed very high effect sizes: VAS pain (r = 0.98; 95% CI [0.97–0.99]), sciatica (r = 0.97; 95% CI [0.95–0.98]), spinal pain (r = 0.98; 95% CI [0.97–0.99]), limb pain (r = 0.97; 95% CI [0.95–0.98]), and limb numbness (r = 0.97; 95% CI [0.95–0.98]), confirming substantial improvements following surgical treatment.

**Figure 2:**
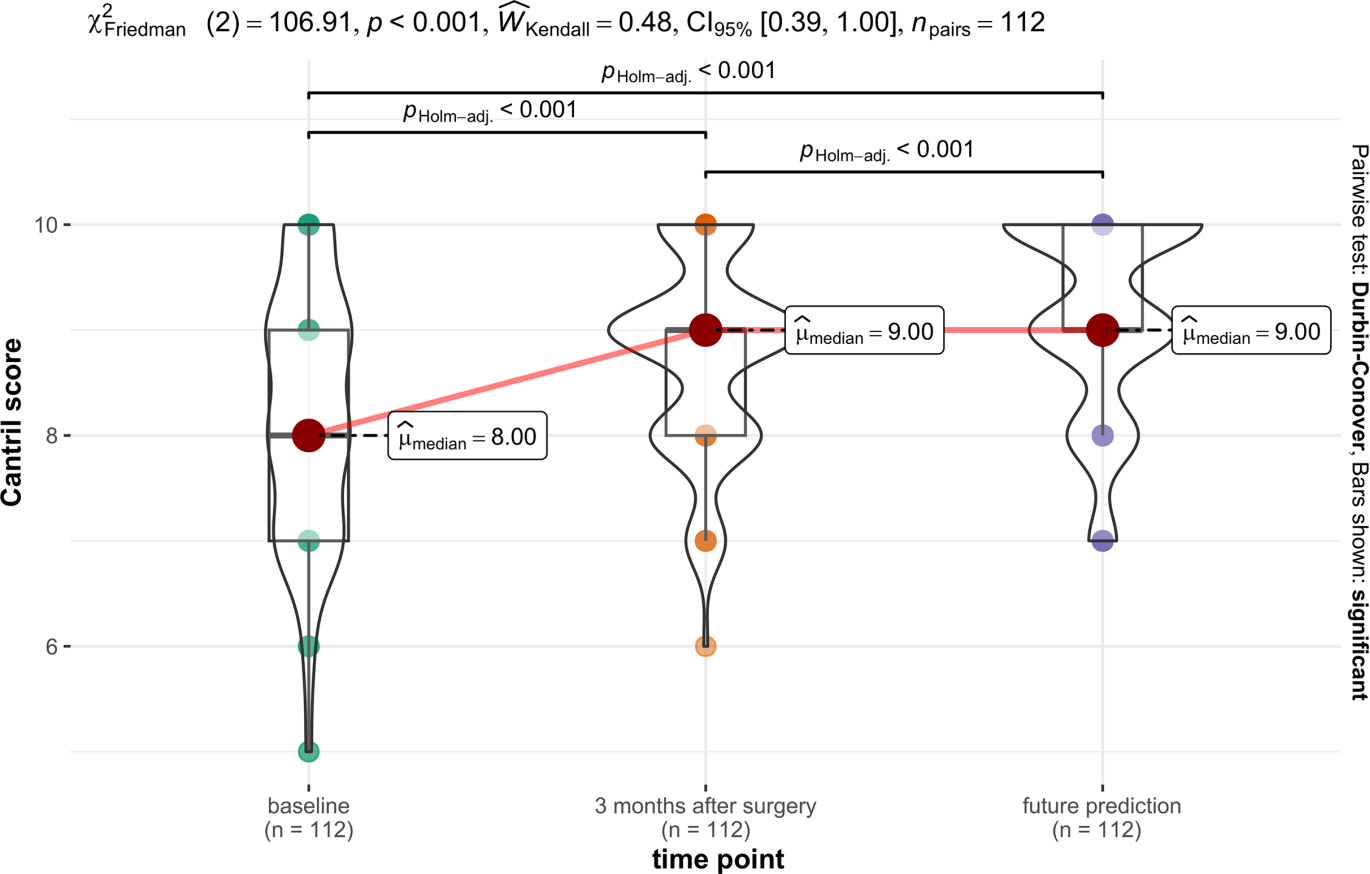
Temporal dynamics of the Cantril score of life satisfaction in patients with discopathy.

Analysis of temporal trend in life satisfaction using the Friedman test showed significant improvement over time (*p* < 0.001, Kendall’s W = 0.58). Median satisfaction before symptom onset was 8.00 (7.00–9.00), at 3 months post-surgery 9.00 (8.00–9.00), and future prognosis 9.00 (9.00–10.00). The large effect size (W = 0.58) indicates a clinically significant change in perceived quality of life. Durbin-Conover *post-hoc* tests with Holm correction confirmed significant differences between all time points, with patients not only recovering their pre-disease level of satisfaction but also predicting even greater improvement in the future, indicating therapeutic optimism. A graphical presentation of life satisfaction dynamics at three time points along with *post-hoc* test results is shown in Fig. 2.

### Observed differences in clinical outcomes between surgical approaches

Important note: The following observations should be interpreted with caution due to non-randomized surgical selection based on clinical indications. Patients selected for PLIF *vs*. ALIF differed in baseline pathology (spinal canal stenosis *vs*. discogenic pain, presence *vs*. absence of posterior compression), which introduces selection bias that cannot be fully controlled through regression analysis. The observed differences may reflect patient characteristics and surgical indications rather than inherent technique superiority. Direct comparison of effectiveness is not possible in this retrospective observational design without randomization.

Regression analysis with interaction terms revealed several differences in clinical outcomes between surgical approaches (Table 4). Patients undergoing ALIF demonstrated different pain reduction patterns compared to PLIF in the 3-month follow-up period. The interaction analysis indicated that ALIF was associated with 1.66 points (95% CI [0.93–2.40], *p* < 0.001) greater reduction in overall VAS pain, 1.44 points (95% CI [0.53–2.34], *p* = 0.002) greater reduction in spinal pain, and 1.41 points (95% CI [0.53–2.29], *p* = 0.002) greater reduction in limb pain compared to PLIF. Baseline characteristics also differed between groups: patients selected for ALIF had higher baseline quality of life (0.66 points, 95% CI [0.28–1.04], *p* = 0.001) and lower baseline limb numbness (1.98 points, 95% CI [0.98–2.97], *p* < 0.001).

**Table 4:** Observed differences in clinical outcomes between PLIF and ALIF surgical approaches–synthesis of regression results.

Clinical parameter	Baseline effect PLIF *vs*. ALIF		Time interaction effect		Observed pattern
	B (95% CI)	*p*	B (95% CI)	*p*	
**Overall pain (VAS)**	−0.11 [−0.66 to 0.44]	0.690	**1.66 [0.93 to 2.40]**	**<0.001**	**ALIF group: 1.66 pts greater reduction**
**Spinal pain (NRS)**	−0.59 [−1.27 to 0.09]	0.090	**1.44 [0.53–2.34]**	**0.002**	**ALIF group: 1.44 pts greater reduction**
**Limb pain (NRS)**	**−0.93 [−1.59 to −0.27]**	**0.006**	**1.41 [0.53–2.29]**	**0.002**	**ALIF group: greater reduction**
**Sciatica (NRS)**	−0.09 [−0.81 to 0.62]	0.795	0.59 [−0.36 to 1.55]	0.222	No significant difference
**Numbness (NRS)**	**1.98 [0.98–2.97]**	**<0.001**	**−1.45 [−2.78 to −0.12]**	**0.032**	**PLIF group: greater reduction**
**Stiffness ** **(NRS)**	**1.55 [0.30–2.79]**	**0.015**	−0.79 [−2.46 to 0.87]	0.349	No significant differences in reduction
**Sensory impairment**	N/A	N/A	N/A	N/A	No significant differences
**Motor deficits**	N/A	N/A	N/A	N/A	No significant differences
**Foot drop**	N/A	N/A	N/A	N/A	No significant differences

**Notes:**

These differences should be interpreted with caution due to non-randomized patient selection based on distinct clinical indications. Baseline differences in pathology, patient characteristics, and surgical indications may contribute to observed outcome patterns independent of surgical technique.

**Baseline effect:** PLIF *vs*. ALIF difference at baseline level; Time interaction effect: differential change at 3 months (3 months × PLIF interaction); N/A: Not applicable (no significant effects in regression models); Model: Robust linear regression (RLM), *n* = 112 patients, 224 observations. Bold values indicate statistically significant results (*p* < 0.05).

Conversely, patients undergoing PLIF demonstrated greater improvement in limb numbness over time (interaction coefficient: −1.45 points, 95% CI [−2.78 to −0.12], *p* = 0.032) and had lower baseline limb pain intensity (0.93 points, 95% CI [0.27–1.59], *p* = 0.006). For sciatica, sensory impairment, motor deficits, and foot drop, regression analysis did not reveal statistically significant differences in outcomes between the surgical approaches.

These observed differences likely reflect both the distinct surgical techniques and the different patient populations selected for each approach based on clinical indications and anatomical considerations. Both surgical methods demonstrated substantial pain reduction and quality of life improvement at 3-month follow-up, though the specific symptom response profiles differed between groups. The differences in baseline characteristics and surgical indications make it impossible to attribute observed outcome differences solely to surgical technique. Given the non-randomized design and inherent selection bias, definitive conclusions regarding relative effectiveness cannot be drawn from these observational data.

Among modifiable factors examined in multifactorial analyses, physical activity was associated with improved quality of life (B = 0.33; 95% CI [0.10–0.57], *p* = 0.006), while tobacco smoking was associated with increased spinal stiffness (B = 1.53; 95% CI [0.73–2.33], *p* < 0.001). Age showed differential associations with individual symptoms, with older age associated with reduced sciatica intensity (B = −0.02 per year; 95% CI [−0.04 to −0.00], *p* = 0.019) but increased spinal pain (B = 0.02 per year; 95% CI [0.00–0.04], *p* = 0.015) and stiffness (B = 0.05 per year; 95% CI [0.02–0.09], *p* = 0.002). Spinal stiffness represented the only symptom without statistically significant improvement in the 3-month observation period (B = −0.56; 95% CI [−1.91 to 0.79], *p* = 0.414), warranting attention in long-term management. The positive relationship between baseline pain intensity and quality of life (B = 0.20; 95% CI [0.10–0.31], *p* < 0.001) may reflect response shift phenomena or the importance of patient expectations and access to surgical care in perceived treatment benefit.

## Discussion

The conducted study provides information on the intensity of pain complaints and evaluation of life satisfaction in patients with lumbar disc disease after surgical treatment using interbody stabilization—PLIF and ALIF. The results confirm that both methods lead to significant reduction in pain intensity and improvement in patients’ quality of life, which is consistent with reports from other authors (Anzai et al., 2024; Tung et al., 2023; Shih et al., 2023; Liu et al., 2014).

In our studies, analysis of pain intensity dynamics showed significant reduction in complaints 3 months after surgery regardless of the method used, confirming substantial pain reduction following surgical treatment. International studies show significant improvement in pain reduction in patients undergoing both methods. PLIF: average VAS improvement from 8.0 points preoperatively to 2.7 points after 2 years of observation. ALIF: similar results with VAS improvement from 8.2 points to 2.4 points in the same period (Tung et al., 2023). Statistically, no significant differences were found between PLIF and ALIF methods in terms of pain reduction during 12 and 24-month observation periods (Anzai et al., 2024).

In our study, we observed an interaction between time after surgery and type of operation, indicating less pain reduction after PLIF compared to ALIF. This may suggest that the ALIF method, although technically more demanding, may provide better effects in pain reduction. Results from other authors were similar. In the latest systematic reviews, ALIF superiority in pain reduction over time was noted. In the Rathbone 2023 study, pain reduction was observed 1 year after surgery comparing ALIF *vs*. PLIF (MD −1.00, CI [−1.47 to −0.53]), while in the second year comparing ALIF *vs*. PLIF (MD −1.39, CI [−1.67 to −1.11]) (Rathbone et al., 2023). Studies indicate several mechanisms explaining better pain reduction in ALIF. ALIF allows for introduction of larger implants, which translates to better load distribution (Scott-Young et al., 2024; Odeh et al., 2020), avoidance of damage to paraspinal muscles and posterior ligaments (Veliky et al., 2024), possibility of direct spinal curvature correction (Tung et al., 2023; Mobbs et al., 2015), more effective indirect decompression through restoration of intervertebral disc height, which leads to widening of intervertebral foramina (Odeh et al., 2020).

Analysis of the influence of various factors on pain intensity and quality of life brought several important observations. In our studies, we noted significant reduction of sciatica symptoms by 7.13 points (baseline result 7.52 points) in the entire study group. Researchers suggest that reduction of sciatica symptoms after surgery is observed most intensively within the first 3 months after surgery regardless of operation type (ALIF/PLIF) (Skolasky et al., 2018). Temporal regressions show that early sciatica improvement strongly correlates with overall improvement in function and quality of life after 1 year.

Analysis of lower limb symptoms (pain, numbness, paresis) confirms that the percentage of patients declaring clear improvement ranges from 75% to 85% for both techniques, with differences between ALIF and PLIF being statistically insignificant—some meta-analysis reviews show slight ALIF superiority in long-term elimination of pain radiation, especially in younger and less advanced patients (Rathbone et al., 2023; Madan & Boeree, 2003). In our studies, as with other authors, numbness decreases significantly after both operations (Skolasky et al., 2018; Mancuso et al., 2017), however, in some patients symptoms persist for 6–12 months. Regression study results suggest that the severity of neurological changes and duration of their presence before surgery are the strongest predictors of complete sensory recovery. In our studies, we observed greater reduction of numbness after PLIF than after ALIF, however, attention should be paid to the fact that before surgery the numbness parameter was higher in PLIF than in ALIF.

Spinal stiffness assessed before and after surgery improved in both types of procedures. According to conducted regression meta-analyses, ALIF may be associated with slightly less subjectively perceived stiffness after surgical intervention, thanks to preservation of posterior muscular and ligamentous structures, however, there are no data on significant clinical differences between techniques. Additionally, emerging evidence suggests relationships between hip and pelvic range of movement and outcomes after lumbar stabilization procedures, as restricted pelvic mobility may compensate for spinal stiffness and influence perceived functional outcomes (Rathbone et al., 2023).

Patients’ quality of life, measured by Cantril’s ladder, showed significant improvement at subsequent time points, reaching the highest values in future prognosis. This indicates patient optimism regarding long-term treatment effects and confirms the importance of surgical intervention not only in pain reduction but also in improving overall well-being. Similar observations were made by other authors (Madan & Boeree, 2003; Juricek et al., 2010). It was also observed that patients after PLIF reported lower baseline quality of life compared to patients after ALIF, which may be related to differences in baseline clinical condition of patients qualified for individual surgical methods. The positive relationship between baseline pain intensity and quality of life represents an interesting observation that may suggest that patients with higher pain intensity may experience greater relief and satisfaction from treatment, which translates to better quality of life assessment. In our studies, similar to other authors (Efendi et al., 2018), we additionally observed that patients who were physically active before surgery had greater improvement in quality of life assessment, which is also confirmed by other researchers (Li et al., 2023; Teng et al., 2017). Similarly, the negative effect of tobacco smoking on spinal stiffness observed by us is consistent with reports on the effect of nicotine on the healing process and bone tissue metabolism. Analysis of 24 comparative studies showed that lifestyle-related factors have greater impact on long-term patient satisfaction than choice of specific surgical technique (Wong et al., 2024; Wang et al., 2023). This is an important observation for clinicians planning a holistic approach to disc disease treatment.

It should be remembered that analysis of factors affecting quality of life after interbody stabilization should be considered in the context of current clinical guidelines. The latest NASS guidelines from 2024 emphasize the importance of multidimensional treatment outcome assessment, taking into account not only pain reduction but also functionality and patient satisfaction. The Enhanced Recovery After Surgery (ERAS) Society consensus from 2021 draws attention to the importance of a holistic approach to perioperative care, which may affect patients’ perception of quality of life (Mobbs et al., 2015; Sauro et al., 2024).

The observed relationship between baseline pain and quality of life (B = 0.20; 95% CI [0.10–0.31], *p* < 0.001) indicates that patients with higher pain intensity before surgery tend to rate their quality of life higher. This result may be related to the “response shift” phenomenon—a well-documented change in internal standards, values, or conceptualization of quality of life in response to health state changes (Sawatzky et al., 2024). Response shift theory posits that individuals recalibrate their expectations and redefine what constitutes satisfactory quality of life when facing chronic illness and treatment prospects. Patients with more severe symptoms may have greater expectations regarding improvement after surgical intervention, which translates to better quality of life assessment in the context of effective treatment availability (Sawatzky et al., 2024).

Beyond the primary surgical outcomes, multifactorial regression analysis examined demographic and behavioral predictors of pain reduction and quality of life improvement. Analysis of the impact of postoperative pain intensity and the degree of its reduction may differ depending on many factors. In our studies, we did not observe the influence of sex, age, tobacco smoking, or performing physical work on pain reduction after surgical intervention. Studies suggest (Parrish et al., 2020; Triebel et al., 2017) that women may report higher levels of preoperative pain and greater disability. However, differences in pain reduction after PLIF or ALIF surgery between sexes are usually statistically insignificant; women and men gain similarly clinically after surgery, although individual pain perception and time to return to activity may differ. Age does not have a clear effect on absolute pain reduction after surgery. Older age is associated with increased risk of complications and longer recovery time, while the direct effect of surgery on pain reduction is similar regardless of age. In some analyses, younger people without comorbidities may recover function faster (Choy et al., 2017). Scientific studies show that tobacco smoking is a factor that negatively affects the outcomes of surgical treatment of spinal pain. Smoking patients more frequently report persistent pain complaints and have worse postoperative results (less improvement and greater risk of complications) (Meester et al., 2025; Koszela & Wołdańska-Okońska, 2021; Andersen et al., 2018). Similarly, people performing heavy physical work may be more prone to recurrence of pain complaints after surgery and slower return to full function (Wu et al., 2023; Hajilo et al., 2024).

### Study strengths and limitations

The study’s strengths include a comprehensive approach to clinical assessment that encompasses not only overall pain intensity on the VAS scale but also analysis of eight disc disease symptoms using the NRS scale, enabling a broad picture of therapeutic effectiveness. Using Cantril’s ladder to measure quality of life at three time points allows assessment not only of the patient’s current condition but also retrospective and prospective well-being analysis. The main study limitation is the 3-month observation period, which—while allowing assessment of early therapeutic effects—does not enable analysis of long-term outcomes. Standards in spinal surgery recommend a minimum 12–24 month follow-up for complete assessment of interbody stabilization procedure effectiveness (Rathbone et al., 2023). However, studies indicate that most clinical benefits from these procedures manifest in the first 3–6 months, and early results are predictive of long-term outcomes. Additionally, uneven group distribution, with PLIF predominance (65.18%) over ALIF (34.82%), may limit statistical power of some intergroup comparisons. The study does not consider technical procedure parameters such as implant type and size, operator experience, or surgical technique details, which may affect clinical outcomes. This should be considered in future studies. Duration of symptoms prior to surgical intervention was not systematically documented in medical records, preventing analysis of this important prognostic factor’s influence on outcomes. This study did not include *a priori* sample size calculation. The sample size was determined by the number of patients meeting inclusion criteria during the study period (2021–2022), which may limit statistical power for detecting smaller effect sizes, particularly in subgroup analyses. An important limitation is the absence of a discectomy comparison group, limiting our ability to establish fusion superiority. Future prospective studies should compare fusion *vs*. discectomy in matched populations with standardized selection criteria, minimum 24-month follow-up, cost-effectiveness analysis, and assessment of fusion rates and adjacent segment disease.

## Conclusions

Early results at 3-month follow-up indicate that both PLIF and ALIF demonstrate potential benefit in reducing pain and improving quality of life in patients with lumbar disc disease. The observed differences in surgical technique selection between sexes highlight the need for better understanding of factors influencing method choice. Modifiable factors including physical activity and tobacco smoking may affect treatment outcomes and should be considered in comprehensive patient management. Spinal stiffness, as the only symptom without significant improvement in the 3-month observation period, requires special attention in long-term management. Prospective studies with patient randomization, standardized selection criteria, and minimum 24-month follow-up are essential for definitive comparison of both methods and to account for the selection bias inherent in retrospective observational designs.

## Supplemental Information

10.7717/peerj.21582/supp-1 Supplemental Information 1 STROBE Checklist.

10.7717/peerj.21582/supp-2 Supplemental Information 2 Raw Data.

10.7717/peerj.21582/supp-3 Supplemental Information 3 Statistical analysis.

## List of abbreviations

ALIF: Anterior Lumbar Interbody Fusion
BMI: Body Mass Index
CI: Confidence Interval
NRS: Numerical Rating Scale
PLIF: Posterior Lumbar Interbody Fusion
VAS: Visual Analogue Scale

## Additional Information and Declarations

### Data Availability

The following information was supplied regarding data availability:

The raw data is available in the Supplemental Files.
