# Supplementary material for: Analysis of pain intensity and quality of life in patients with lumbar disc disease treated with PLIF (Posterior Lumbar Interbody Fusion) and ALIF (Anterior Lumbar Interbody Fusion) interbody stabilization methods: a 3-month retrospective analysis
Source: PeerJ. 2026 Aug 6;14:e21582. doi: 10.7717/peerj.21582 (PMC13453140; doi:10.7717/peerj.21582)
Supplement: Supplementary file 1 [file peerj-14-21582-s001.docx]

STROBE Checklist - Observational Study Reporting Standards

STROBE Statement—checklist of items that should be included in reports of observational studies

|  | Item No | Recommendation | Page  No | Comments/Location in Manuscript |
| --- | --- | --- | --- | --- |
| **Title and abstract** | 1 | (*a*) Indicate the study’s design with a commonly used term in the title or the abstract | 1 | "Retrospective Analysis" clearly stated in title |
|  |  | (*b*) Provide in the abstract an informative and balanced summary of what was done and what was found | 1 | Structured abstract includes Design, Setting, Participants, Main Outcome Measures, Results, and Conclusions |
| Introduction | | | |  |
| Background/rationale | 2 | Explain the scientific background and rationale for the investigation being reported | 2-3 | Background on lumbar disc disease prevalence, treatment methods, and need for comparative analysis |
| Objectives | 3 | State specific objectives, including any prespecified hypotheses | 3 | "The aim of this study was to analyze the intensity of pain complaints and evaluate life satisfaction..." |
| Methods | | | |  |
| Study design | 4 | Present key elements of study design early in the paper | 4 | "A retrospective observational cohort study was conducted..." |
| Setting | 5 | Describe the setting, locations, and relevant dates, including periods of recruitment, exposure, follow-up, and data collection | 4 | Regional Specialist Hospital in Słupsk, Poland; patients treated between 2021-2022; 3-month follow-up |
| Participants | 6 | (*a*) *Cohort study*—Give the eligibility criteria, and the sources and methods of selection of participants. Describe methods of follow-up  *Case-control study*—Give the eligibility criteria, and the sources and methods of case ascertainment and control selection. Give the rationale for the choice of cases and controls  *Cross-sectional study*—Give the eligibility criteria, and the sources and methods of selection of participants | 4 | Inclusion/exclusion criteria clearly stated; retrospective analysis of medical records |
|  |  | (*b*) *Cohort study*—For matched studies, give matching criteria and number of exposed and unexposed  *Case-control study*—For matched studies, give matching criteria and the number of controls per case | N/A | Not applicable - not a matched study |
| Variables | 7 | Clearly define all outcomes, exposures, predictors, potential confounders, and effect modifiers. Give diagnostic criteria, if applicable | 4-5 | Primary outcomes: VAS pain scale, NRS for specific symptoms, Cantril's ladder for QoL. Exposures: PLIF vs ALIF procedures |
| Data sources/ measurement | 8* | For each variable of interest, give sources of data and details of methods of assessment (measurement). Describe comparability of assessment methods if there is more than one group | 4-5 | Data sources described: hospital medical records, preoperative assessments, surgical protocols, follow-up documentation |
| Bias | 9 | Describe any efforts to address potential sources of bias | 5-6 | Robust linear regression with M-estimator used to handle outliers; standardized assessment tools |
| Study size | 10 | Explain how the study size was arrived at | 4 | All patients meeting inclusion criteria in the specified time period were included (convenience sampling) |
| Quantitative variables | 11 | Explain how quantitative variables were handled in the analyses. If applicable, describe which groupings were chosen and why | 5-6 | Normality testing with Shapiro-Wilk; appropriate statistical tests chosen; age and BMI centered relative to median |
| Statistical methods | 12 | (*a*) Describe all statistical methods, including those used to control for confounding | 5-6 | Robust linear regression with multiple predictors; interaction analysis between time and procedure type |
|  |  | (*b*) Describe any methods used to examine subgroups and interactions | 5-6 | Interaction analysis between time point and procedure type (PLIF vs ALIF) |
|  |  | (*c*) Explain how missing data were addressed | 5 | Case-by-case exclusion mentioned; complete documentation requirement in inclusion criteria |
|  |  | (*d*) *Cohort study*—If applicable, explain how loss to follow-up was addressed  *Case-control study*—If applicable, explain how matching of cases and controls was addressed  *Cross-sectional study*—If applicable, describe analytical methods taking account of sampling strategy | 4 | Complete documentation at required time points was inclusion criterion |
|  |  | (*e*) Describe any sensitivity analyses | 5-6 | Robust regression methods used as sensitivity approach to handle outliers |

Continued on next page

| Results | | | |  |
| --- | --- | --- | --- | --- |
| Participants | 13* | (a) Report numbers of individuals at each stage of study—eg numbers potentially eligible, examined for eligibility, confirmed eligible, included in the study, completing follow-up, and analysed | 6 | 112 patients total: 73 PLIF (65.18%), 39 ALIF (34.82%) |
|  |  | (b) Give reasons for non-participation at each stage | 4 | Exclusion criteria: unavailability of data at required time points, incomplete documentation |
|  |  | (c) Consider use of a flow diagram | N/A | Flow diagram not included |
| Descriptive data | 14* | (a) Give characteristics of study participants (eg demographic, clinical, social) and information on exposures and potential confounders | 6-7 | Table 1 provides comprehensive demographic characteristics by sex and overall |
|  |  | (b) Indicate number of participants with missing data for each variable of interest | 6 | Complete data availability was inclusion criterion |
|  |  | (c) *Cohort study*—Summarise follow-up time (eg, average and total amount) | 4 | 3-month follow-up period clearly stated |
| Outcome data | 15* | *Cohort study*—Report numbers of outcome events or summary measures over time | *7-11* | Pain scores at baseline and 3 months; QoL at three time points |
|  |  | *Case-control study—*Report numbers in each exposure category, or summary measures of exposure | *N/A* |  |
|  |  | *Cross-sectional study—*Report numbers of outcome events or summary measures | *N/A* |  |
| Main results | 16 | (*a*) Give unadjusted estimates and, if applicable, confounder-adjusted estimates and their precision (eg, 95% confidence interval). Make clear which confounders were adjusted for and why they were included | 7-11 | Tables 2-4 provide regression coefficients with 95% CI; adjusted for age, sex, BMI, smoking, physical activity |
|  |  | (*b*) Report category boundaries when continuous variables were categorized | 6-7 | Age categories, BMI categories clearly defined in Table 1 |
|  |  | (*c*) If relevant, consider translating estimates of relative risk into absolute risk for a meaningful time period | 7-11 | Effect sizes and clinical significance discussed |
| Other analyses | 17 | Report other analyses done—eg analyses of subgroups and interactions, and sensitivity analyses | 11 | Interaction analysis, post-hoc tests with multiple comparison correction |
| Discussion | | | |  |
| Key results | 18 | Summarise key results with reference to study objectives | 12-13 | Results summarized in relation to stated objectives |
| Limitations | 19 | Discuss limitations of the study, taking into account sources of potential bias or imprecision. Discuss both direction and magnitude of any potential bias | 16 | Comprehensive limitations section addressing study period, group distribution, technical parameters |
| Interpretation | 20 | Give a cautious overall interpretation of results considering objectives, limitations, multiplicity of analyses, results from similar studies, and other relevant evidence | 12-16 | Discussion includes comparison with other studies and cautious interpretation |
| Generalisability | 21 | Discuss the generalisability (external validity) of the study results | 16-17 | Generalizability discussed in context of single-center study and patient population |
| Other information | | | |  |
| Funding | 22 | Give the source of funding and the role of the funders for the present study and, if applicable, for the original study on which the present article is based | Title page | "This research received no external funding" stated in acknowledgments |

*Give information separately for cases and controls in case-control studies and, if applicable, for exposed and unexposed groups in cohort and cross-sectional studies.

**Note:** An Explanation and Elaboration article discusses each checklist item and gives methodological background and published examples of transparent reporting. The STROBE checklist is best used in conjunction with this article (freely available on the Web sites of PLoS Medicine at http://www.plosmedicine.org/, Annals of Internal Medicine at http://www.annals.org/, and Epidemiology at http://www.epidem.com/). Information on the STROBE Initiative is available at www.strobe-statement.org.
