# Supplementary material for: Analysis of pain intensity and quality of life in patients with lumbar disc disease treated with PLIF (Posterior Lumbar Interbody Fusion) and ALIF (Anterior Lumbar Interbody Fusion) interbody stabilization methods: a 3-month retrospective analysis
Source: PeerJ. 2026 Aug 6;14:e21582. doi: 10.7717/peerj.21582 (PMC13453140; doi:10.7717/peerj.21582)
Supplement: Supplementary file 2 [file peerj-14-21582-s003.docx]

# 1. Materials and methods

Statistical analysis

In this analysis, the threshold for statistical significance was established at an alpha level of 0.05. The normality of the numeric variables was assessed using the Shapiro-Wilk test. For distributions that deviated from normality, the data were described using the median (*Mdn*) and interquartile range, specifically the first (*Q1*) and third (*Q3*) quartiles. For categorical variables, the group size (*n*) and percentage of each category were reported.

For comparing two independent groups with numerical variables that exhibited non-normal distributions, the Wilcoxon rank sum test was employed. When assessing within-group differences across two time points, the Wilcoxon signed-rank test was utilized. The effect size for these analyses was quantified using a rank-based two-point correlation coefficient.

In cases where within-group differences across three time points were analyzed and where the distributions deviated from normality, the Friedman test was applied. Subsequent analyses were conducted using Durbin-Conover post-hoc tests. To adjust for the increased risk of type I errors due to multiple comparisons, p-values were corrected using Holm's method. Kendall's W was calculated as the measure of effect size for these comparisons.

The estimation of the effects of multiple factors on the outcome variables was conducted using multiple linear regression, employing the robust M estimator through iterated re-weighted least squares (IWLS) methodology. This approach, known as robust linear modeling (RLM), is particularly feasible in scenarios where there is non-normality of residuals and heteroscedasticity, effectively addressing these common issues in regression analysis.

Characteristics of the statistical tool

Analyses were conducted using the R Statistical language (version 4.3.1; R Core Team, 2023) on Windows 10 pro 64 bit (build 19045), using the packages *emmeans* (version 1.8.9; Lenth R, 2023), *sjPlot* (version 2.8.15; Lüdecke D, 2023), *parameters* (version 0.21.3; Lüdecke D et al., 2020), *performance* (version 0.10.8; Lüdecke D et al., 2021), *report* (version 0.5.7; Makowski D et al., 2023), *ggstatsplot* (version 0.12.1; Patil I, 2021), *gtsummary* (version 1.7.2; Sjoberg D et al., 2021), *MASS* (version 7.3.60; Venables WN, Ripley BD, 2002), *readxl* (version 1.4.3; Wickham H, Bryan J, 2023) and *dplyr* (version 1.1.3; Wickham H et al., 2023).

Characteristics of the sample

The analysis encompassed the results from 112 patients diagnosed with disc disease, ranging in age from 17 to 72 years, including 64 women (57.1%) and 48 men (42.9%). The factors detailed in Table 1 are essential for comprehensively understanding the broader health landscape associated with discopathy.

**Table 1.** Demographic, lifestyle, comorbidity, employment, and work type characteristics of the overall sample of discopathy patients and stratified by sex*.*

| *Characteristic* | *N* | *Overall sample ^a^* | *sex* | | *p^c^* |
| --- | --- | --- | --- | --- | --- |
|  |  |  | *female, n = 64^a^* | *male, n = 48^a^* |  |
| *Demographic* | | | | | |
| Age, years | 112 | 43.00  (36.00, 55.00)*^b^* | 45.00  (36.75, 56.00) *^b^* | 43.00  (35.00, 52.00) *^b^* | 0.497*^d^* |
| age (categories) | 112 |  |  |  | 0.601 |
| ≤ 30 yrs. |  | 16.00 (14.29%) | 7.00 (10.94%) | 9.00 (18.75%) |  |
| 31-45 yrs. |  | 48.00 (42.86%) | 27.00 (42.19%) | 21.00 (43.75%) |  |
| 46-60 yrs. |  | 36.00 (32.14%) | 23.00 (35.94%) | 13.00 (27.08%) |  |
| ≥ 61 yrs |  | 12.00 (10.71%) | 7.00 (10.94%) | 5.00 (10.42%) |  |
| BMI, kg/m^2^ | 112 | 27.00  (23.00, 30.00) *^b^* | 26.00  (23.00, 29.25) *^b^* | 27.00  (24.00, 31.00) *^b^* | 0.394 *^d^* |
| BMI type | 112 |  |  |  | 0.216*^e^* |
| normal |  | 39.00 (34.82%) | 26.00 (40.63%) | 13.00 (27.08%) |  |
| overweight |  | 43.00 (38.39%) | 23.00 (35.94%) | 20.00 (41.67%) |  |
| obesity (I degree) |  | 28.00 (25.00%) | 13.00 (20.31%) | 15.00 (31.25%) |  |
| obesity (II degree) |  | 2.00 (1.79%) | 2.00 (3.13%) | 0.00 (0.00%) |  |
| *Lifestyle and comorbidities* | | | | | |
| smoking | 112 | 55.00 (49.11%) | 31.00 (48.44%) | 24.00 (50.00%) | 0.870 |
| hypothyroidism | 112 | 18.00 (16.07%) | 13.00 (20.31%) | 5.00 (10.42%) | 0.158 |
| HT | 112 | 14.00 (12.50%) | 10.00 (15.63%) | 4.00 (8.33%) | 0.248 |
| DM | 112 | 3.00 (2.68%) | 1.00 (1.56%) | 2.00 (4.17%) | 0.575 *^e^* |
| other | 112 | 4.00 (3.57%) | 3.00 (4.69%) | 1.00 (2.08%) | 0.634 *^e^* |
| *Employment and type of work performed* | | | | | |
| working | 112 | 83.00 (74.11%) | 45.00 (70.31%) | 38.00 (79.17%) | 0.290 |
| physical | 112 | 69.00 (61.61%) | 37.00 (57.81%) | 32.00 (66.67%) | 0.340 |
| white-collar job | 112 | 8.00 (7.14%) | 3.00 (4.69%) | 5.00 (10.42%) | 0.285 *^e^* |
| other type job | 112 | 6.00 (5.36%) | 5.00 (7.81%) | 1.00 (2.08%) | 0.235 *^e^* |
| *^a^*  *n* (%)  *^b^* *Mdn* (*Q1*, *Q3*)  *^c^*  Pearson’s Chi-squared test  *^d^*  Wilcoxon rank sum test  *^e^*  Fisher’s exact test | | | | | |

The median age of the cohort was 43.0 years, with a slightly older median age observed in females (45.0) compared to males (43.0). The age distribution highlights a predominance in the middle-age categories, which may suggest a peak in vulnerability or diagnosis during these years. The BMI data, with a median value of 27 kg/m^2^, indicates that the majority of the patient population falls within the overweight category. This is particularly pronounced in the male subset, where a higher proportion of individuals are classified under obesity (I degree) compared to females. The gender-stratified analysis revealed no significant differences in BMI categories between males and females, which may indicate that its impact is consistent across genders.

Smoking, which is prevalent in 49.11% of the patient cohort, shows a near-equal distribution between females and males, suggesting that smoking as a risk factor for discopathy is equally significant across genders.

Hypothyroidism was found in 16.07% of the total cohort, with a higher incidence among females (20.31%) compared to males (10.42%). This gender disparity could indicate a potential hormonal or genetic predisposition among females that may exacerbate or influence the development of discopathy.

Hypertension (HT) was present in 12.50% of the patients, with a higher representation again in females (15.63%) than in males (8.33%). This observation might suggest that females with discopathy could be at a greater risk of cardiovascular comorbidities, or alternatively, that hypertension could contribute to the progression of disc disease more prominently in females.

Diabetes Mellitus (DM) appeared in a relatively small proportion of the sample (2.68%), with a slightly higher occurrence in males (4.17%) compared to females (1.56%). Although the numbers are small, the presence of DM in the discopathy patient cohort is a critical factor, as diabetes can affect disease outcomes through microvascular changes, which might complicate the clinical management of discopathy.

Other comorbidities were noted in 3.57% of the patients, with a minor gender variation, which seems to have less clinical significance.

A substantial 74.11% of the cohort maintain active employment despite their condition with a higher percentage of males (79.17%) working compared to females (70.31%).

Physical labor is the predominant type of work among the patients, involving 61.61% of the sample. The distribution leans more heavily towards males (66.67%) compared to females (57.81%). The high prevalence of physical work among discopathy patients, particularly in males, supports the hypothesis that physically demanding occupations might contribute to either the onset or exacerbation of discopathy symptoms.

Only a small fraction of the cohort is engaged in white-collar jobs, with 7.14% overall, where males again are slightly more represented (10.42%) than females (4.69%). This could suggest that individuals in less physically demanding roles are less likely to suffer from discopathy, although the small numbers require cautious interpretation.

The category labeled as 'other types of jobs' is the least common, consisting of 5.36% of the population, with more females (7.81%) than males (2.08%) represented.

# 2. Results

## 2.1 Neurological, pain and quality of life assessment in discopathy patients

The analysis of neurological assessments prior to surgery in table 2 reveals substantial insights into the manifestation and severity of this condition across different genders

**Table 2.** Neurological assessments of discopathy before surgery

| *Characteristic* | *N* | *Overall sample* | *sex* | | *p^b^* |
| --- | --- | --- | --- | --- | --- |
|  |  |  | *female, n = 64^a^* | *male, n = 48^a^* |  |
| surgery type: | 112 |  |  |  | **< 0.001** |
| ALIF |  | 39.00 (34.82%) | 33.00 (51.56%) | 6.00 (12.50%) |  |
| PLIF |  | 73.00 (65.18%) | 31.00 (48.44%) | 42.00 (87.50%) |  |
| movement impairments | 112 | 9.00 (8.04%) | 4.00 (6.25%) | 5.00 (10.42%) | 0.494*^c^* |
| sensory impairments | 112 | 14.00 (12.50%) | 7.00 (10.94%) | 7.00 (14.58%) | 0.564 |
| numbness in limbs | 112 | 72.00 (64.29%) | 37.00 (57.81%) | 35.00 (72.92%) | 0.099 |
| spine pain | 112 | 98.00 (87.50%) | 57.00 (89.06%) | 41.00 (85.42%) | 0.564 |
| lower limb pain | 112 | 85.00 (75.89%) | 51.00 (79.69%) | 34.00 (70.83%) | 0.278 |
| sphincter dysfunctions | 112 | 5.00 (4.46%) | 4.00 (6.25%) | 1.00 (2.08%) | 0.390*^c^* |
| *^a^* *n* (%)  *^b^* Pearson’s Chi-squared test  *^c^*  Fisher’s exact test | | | | | |

Notably, the type of surgery significantly varied between males and females, with Anterior Lumbar Interbody Fusion (ALIF) being predominantly performed on females (51.56%) compared to males (12.50%). Conversely, Posterior Lumbar Interbody Fusion (PLIF) was more common in males (87.50%) than in females (48.44%). These findings, distinguished by a highly significant p-value (*p* < 0.001), suggest that the anatomical and perhaps pathological differences between genders may influence the surgical approach and strategy in discopathy treatment.

Regarding neurological impairments, movement impairments were observed in 8.04% of the overall sample with a slightly higher prevalence in males (10.42%) than in females (6.25%), although this difference was not statistically significant. Similarly, sensory impairments were reported in 12.50% of patients, with a balanced occurrence between males and females, further implying that sensory deficits due to discopathy do not significantly differ by gender.

A notable disparity was observed in the prevalence of numbness in limbs, affecting 64.29% of the cohort, with a higher incidence in males (72.92%) compared to females (57.81%). This may indicate a more severe or different pattern of nerve involvement in males, although the p-value of 0.099 was only on the trend level.

Spine pain and lower limb pain were the most common symptoms, reported in 87.50% and 75.89% of patients respectively, underscoring the profound impact of discopathy on pain perception and physical function. The slight differences in the prevalence of these symptoms between genders were not statistically significant, indicating that both sexes experience substantial pain and discomfort from discopathy.

Sphincter dysfunctions, although less common, were present in 4.46% of the sample, with a higher occurrence in females (6.25%) compared to males (2.08%). This could suggest a differential impact of discopathy on autonomic nerve function between genders, yet the small numbers and a p-value of 0.390 do not lend strong support to this observation.

The data from Table 3 provides an in-depth analysis of the temporal dynamics of pain levels and the severity of neurological symptoms in discopathy patients before and three months after surgical intervention.

**Table 3.** Temporal dynamics of pain levels and severity of neurological symptoms in discopathy patients

| *Characteristic* | *n_pairs_* | *Time point* | | *p^b^* | $\hat{r}_{biserial}^{rank}$ |
| --- | --- | --- | --- | --- | --- |
|  |  | *baseline^a^* | *3 months after surgery^a^* |  |  |
| VAS score | 112 | 8.00 (7.00, 9.00) | 3.50 (2.00, 4.00) | **< 0.001** | 1.00 |
| NRS sciatica | 112 | 8.00 (6.75, 9.00) | 0.00 (0.00, 2.00) | **< 0.001** | 1.00 |
| NRS spinal pain | 112 | 8.00 (7.00, 9.00) | 4.00 (2.00, 5.00) | **< 0.001** | 0.97 |
| NRS limb pain | 112 | 8.00 (7.00, 9.00) | 0.00 (0.00, 2.00) | **< 0.001** | 1.00 |
| NRS numbness | 112 | 7.00 (0.00, 8.00) | 0.00 (0.00, 2.00) | **< 0.001** | 1.00 |
| NRS spinal stiffness | 112 | 0.00 (0.00, 7.00) | 0.00 (0.00, 4.00) | **< 0.001** | 1.00 |
| NRS weakened sensation of the limbs | 112 | 0.00 (0.00, 0.00) | 0.00 (0.00, 0.00) | **< 0.001** | 1.00 |
| NRS motor impairments | 112 | 0.00 (0.00, 0.00) | 0.00 (0.00, 0.00) | **0.022** | 1.00 |
| NRS drop foot | 112 | 0.00 (0.00, 0.00) | 0.00 (0.00, 0.00) | 0.181 | 1.00 |
| *^a^* *Mdn* (*Q1*, *Q3*);  *^b^* Wilcoxon signed rank test | | | | | |

Initially, the VAS for overall pain presented with a median score of 8.00, indicative of high pain levels. Post-surgery, this score significantly reduced to a median of 3.50, highlighting a major alleviation of pain experienced by the patients. This transition suggests effective pain management through surgery.

Further granularity is observed in the Numeric Rating Scale (NRS) assessments for specific types of pain and symptoms. For instance, the NRS for sciatica, which originally had a median score close to the maximum pain level at 8.00, dropped dramatically to zero in most cases post-surgery. This suggests that the surgical intervention was particularly successful in targeting and mitigating the radicular pain commonly associated with sciatica.

Similarly, limb pain, another significant component of discopathy-related suffering, was also noted to have a median score of 8.00 pre-surgery, which then reduced to non-existent or minimal levels (median of 0.00) post-surgery. This substantial reduction underscores the surgery's capacity to alleviate pain that extends beyond the spinal region, addressing peripheral symptoms effectively.

Numbness, a direct indicator of neurological impact, showed a significant decrease from a broad range of severity (median of 7.00) to minimal or no numbness (median of 0.00) post-surgery. This reduction is particularly noteworthy as it reflects the restoration of nerve function or the successful decompression of affected nerves.

While the scores for spinal stiffness, weakened sensation of the limbs, motor impairments, and drop foot were predominantly zero at both time points, suggesting a non-significant presence or change through surgical intervention, the statistical significance observed in these categories likely captures minimal but consistent variations that are clinically negligible.

See Figures from A.1 to A.9 in the Appendices section for a visualization of the results.

The data presented in Table 4 regarding the dynamics of life satisfaction among discopathy patients as measured by the Cantril score over three distinct time points—baseline, three months after surgery, and a future projection—provides crucial insights into the psychosocial impact of discopathy and its treatment.

**Table 4.** Dynamics of level of satisfaction with life in discopathy patients over time

| *Characteristic* | *n_pairs_* | *Time point* | | | *p^b^* |
| --- | --- | --- | --- | --- | --- |
|  |  | *baseline^a^* | *3 months after surgery^a^* | *future prediction^a^* |  |
| Cantril score | 112 | 8.00 (7.00, 9.00) | 9.00 (8.00, 9.00) | 9.00 (9.00, 10.00) | **< 0.001** |
| *^a^* *Mdn* (*Q1*, *Q3*);  *^b^* Kruskal-Wallis rank sum test | | | | | |

At baseline, prior to undergoing surgery, the median Cantril score was registered at 8.00, with the first and third quartiles ranging from 7.00 to 9.00. This indicates a relatively high level of life satisfaction despite the presence of discopathy, possibly reflecting patients' optimism about the forthcoming surgical treatment or their ability to cope with their condition.

Significantly, three months following surgical intervention, there was a notable increase in the Cantril score, with the median rising to 9.00 and the interquartile range tightening to 8.00 to 9.00. This improvement suggests that the surgical treatment not only alleviated physical symptoms, as previously discussed in relation to pain and neurological impairment, but also had a positive impact on the patients' overall perception of their quality of life. The increase in life satisfaction shortly after surgery likely reflects immediate post-operative benefits such as reduced pain and enhanced mobility, which contribute to improved daily functioning and well-being.

Looking ahead, the future predictions for the Cantril score are even more optimistic, with a median expected to reach 9.00 and potentially up to 10.00, indicating near-maximum life satisfaction. This forward-looking estimate underscores the sustained benefits of the surgical intervention, projecting that the initial improvements in health and well-being can lead to long-term positive outcomes in life satisfaction. This projection might also reflect patients' expectations of continued recovery and a return to normalcy or near-normalcy in their lives.

The statistical significance between all changes was highly significant (*p_adj_* < 0.001) due to results of post hoc test, that confirms that the observed improvements and projections are not due to random variation but represent genuine shifts in the life satisfaction of this patient cohort. This robust statistical result strengthens the case for surgical intervention as a highly beneficial treatment modality for discopathy not only in terms of physical health but also in enhancing psychological and overall life satisfaction. See Figure from A.10 in the Appendices section for a visualization of the results with post-hoc tests.

## 2.2 Analyzing the impact of demographics, smoking, work type, and surgery type on overall vas pain intensity on a VAS scale in discopathy patients

The robust linear regression analysis presented in Table B.1 in the Appendices section evaluates the influence of several variables—including demographics, smoking status, work type, and surgery type—on the VAS pain intensity scores among discopathy patients.

From the data, the most compelling finding is the dramatic reduction in VAS pain scores following surgery, with the coefficient for time (three months after surgery) showing a significant decrease of -6.07 points (*95% CI*: -6.67 to -5.48, *p* < 0.001). This indicates a substantial improvement in pain levels post-surgery, underscoring the efficacy of surgical intervention in managing discopathy-related pain.

Regarding demographic factors, neither sex nor age shows a significant impact on the pain scores. The regression coefficient for males compared to the reference group (females) was -0.15 (*95% CI*: -0.54 to 0.23, *p* = 0.434), and the effect of age per year, centered around the median age of 43 years, was minimal and statistically insignificant (*B* = 0.01, *95% CI*: -0.00 to 0.03, p = 0.103).

Similarly, lifestyle and occupational factors such as smoking and engaging in physical work did not demonstrate a significant influence on VAS scores. Smokers showed a slight but not statistically significant increase in pain scores compared to non-smokers (*B* = 0.18*, 95% CI*: -0.17 to 0.53, *p* = 0.313), and those engaged in physical work also showed a negligible difference in pain scores (*B* = 0.03, 95% CI: -0.34 to 0.39, *p* = 0.882).

In terms of surgical type, when comparing PLIF to ALIF as the reference, there was no significant difference in pain outcomes at baseline (B = -0.11, *95% CI*: -0.66 to 0.44, *p* = 0.690). However, an intriguing interaction was observed between time (three months post-surgery) and surgery type (PLIF), where the combination of PLIF surgery and the time effect post-surgery resulted in an additional increase in VAS scores (*B* = 1.66, *95% CI*: 0.93 to 2.40, *p* < 0.001). This suggests that while both surgical types are effective in reducing pain, patients undergoing PLIF might experience a slightly less pronounced reduction in pain or a delayed recovery compared to those undergoing ALIF.

## 2.3 Assessing the effects of demographics, smoking, work type, and surgery type on NRS pain intensity for specific discopathy symptoms

### 2.3.2 NRS for sciatica as the outcome

The robust linear regression analysis focusing on the NRS score for sciatica as the outcome provided key insights into the effectiveness of surgical interventions and other factors influencing sciatica pain. Initially, patients presented with a high baseline sciatica pain score of 7.52 (*CI 95*%: 6.86 – 8.19, *p* < 0.001), stressing the significant burden of sciatica before any treatment was administered.

Gender did not significantly impact the sciatica pain outcomes, as indicated by the minimal and statistically insignificant difference in pain scores between males and females (*B* = 0.02, *CI 95*%: -0.49 – 0.53, *p* = 0.944). This suggests that biological sex does not require consideration for differential treatment strategies in sciatica pain management.

Age showed a slight but statistically significant negative association with sciatica pain, decreasing by 0.02 points for each additional year (*CI 95*%: -0.04 – -0.00, *p* = 0.019). This indicates that older patients might experience a minor reduction in sciatica pain, possibly due to varying pain perception or degenerative changes over time.

BMI did not significantly influence sciatica pain scores, with a coefficient of 0.03 (*CI 95%*: -0.03 – 0.08, *p* = 0.310). This indicates that within the median range, BMI does not play a crucial role in sciatica pain severity.

Smoking status showed a tendency towards higher pain scores in smokers, though this increase was not statistically significant (*B* = 0.33, *CI 95*%: -0.13 – 0.79, *p* = 0.154). This suggests that while smoking might influence pain perception or inflammation, the effect is not pronounced enough to reach statistical significance in this dataset.

Physical work did not significantly affect the pain scores, with a coefficient of -0.10 (*CI 95%*: -0.58 – 0.38, *p* = 0.684). This implies that engagement in physical work does not substantially alter the severity of sciatica pain post-treatment.

The analysis confirmed a significant reduction in sciatica pain scores three months post-surgery, with a decrease of 7.13 points (*CI 95*%: -7.90 – -6.36, *p* < 0.001). This substantial reduction highlights the effectiveness of surgical treatment in alleviating the burden of sciatica.

Regarding the type of surgery, there was no significant difference in baseline pain severity between ALIF and (PLIF, with PLIF showing a minor and statistically insignificant decrease of 0.09 points compared to ALIF (*CI 95*%: -0.81 – 0.62, *p* = 0.795). Additionally, the interaction term between time and surgery type (PLIF) did not significantly influence pain outcomes, with a coefficient of 0.59 (*CI 95*%: -0.36 – 1.55, *p* = 0.222), suggesting that the type of surgical intervention may not significantly impact the long-term outcome in sciatica pain reduction.

### 2.3.2 NRS for spinal pain as the outcome

The analysis detailed in Table B.3 in Appendices section investigates the effects of various demographic variables, lifestyle factors, and surgical types on the NRS for spinal pain in patients with discopathy.

The model's intercept suggests a high baseline spinal pain score of 8.29 (95% CI: 7.66 – 8.92, *p* < 0.001), indicative of the severe discomfort faced by patients prior to any intervention.

The most profound effect on pain reduction is attributed to the time elapsed since surgery, with a significant decrease in NRS pain score of -5.18 (*95% CI*: -5.92 to -4.45, *p* < 0.001) three months post-surgery. This highlights the efficacy of surgical intervention in substantially alleviating spinal pain. Additionally, an interaction effect between time (three months post-surgery) and the type of surgery PLIF shows a 1.44 less decrease in pain scores (*95% CI*: 0.53 to 2.34, *p* = 0.002) compared to ALIF. This suggests that although PLIF is effective in reducing pain, when compared to ALIF, it may not be as effective or might involve a slower recovery trajectory concerning spinal pain relief.

The regression analysis indicates that sex does not significantly influence spinal pain scores, with males showing a negligible and statistically insignificant difference from females (*B* = -0.03, *95% CI*: -0.51 to 0.45, *p* = 0.910). Age, however, does show a slight but significant effect, with each additional year increasing the pain score by 0.02 points (*95% CI*: 0.00 to 0.04, *p* = 0.015). This suggests that older patients might experience slightly higher levels of spinal pain, potentially due to age-related degenerative changes.

Smoking and physical work do not show a statistically significant impact on spinal pain scores, though trends are visible. Smokers tend to report slightly higher pain scores compared to non-smokers, albeit not significantly (*B* = 0.33, *95% CI*: -0.10 to 0.77, *p* = 0.133). Similarly, those engaged in physical work showed a trend towards lower pain scores, though also not reaching statistical significance (*B* = -0.32, *95% CI*: -0.78 to 0.13, *p* = 0.160).

BMI centered around the median value does not significantly affect the NRS spinal pain scores (*B* = 0.01, 95% CI: -0.04 to 0.06, *p* = 0.700), suggesting that within the range of observed BMI values, weight does not play a critical role in modulating spinal pain in discopathy patients.

### 2.3.3 NRS for limb pain as the outcome

The robust linear regression analysis presented regarding the effects of various demographic variables, lifestyle factors, and surgical types on the NRS for spinal pain in discopathy patients reveals several critical insights into the management and prognosis of limb pain post-surgery.

Starting with the intercept at 7.72 (*CI 95%*: 7.11 – 8.33, *p* < 0.001), the high initial NRS limb pain score illustrates the severity of pain experienced by patients prior to any intervention.

The effect of patient sex on pain outcomes revealed no significant difference between males and females, with males having a slight increase in pain score of 0.11 (*CI 95%*: -0.36 – 0.58, *p* = 0.639). This suggests that gender may not play a critical role in the variation of pain scores post-treatment.

Age shows a minor yet statistically significant correlation with pain, with a coefficient of 0.02 (*CI 95%*: 0.00 – 0.04, *p* = 0.032) for each additional year. This indicates that older patients might experience slightly higher pain scores, a factor that should be considered in personalized pain management strategies.

BMI, centered at the median of 27.0 kg/m^2^, did not significantly impact the pain scores, with a coefficient of 0.01 (*CI 95%*: -0.04 – 0.05, *p* = 0.795). This suggests that BMI within the median range does not significantly alter pain outcomes post-surgery.

Smoking status showed a trend toward higher pain scores in smokers compared to non-smokers, though not reaching statistical significance, with a coefficient of 0.40 (*CI 95%*: -0.02 – 0.82, *p* = 0.065). This could indicate a potential aggravation of pain among smokers, warranting further investigation.

Engagement in physical work also showed a trend similar to smoking, with those engaged in physical work reporting slightly higher pain scores, though this was also not statistically significant (*B* = 0.43, *CI 95*%: -0.01 – 0.87, *p* = 0.054).

A significant reduction in pain scores was observed three months post-surgery, with a decrease of 7.78 points (*CI 95*%: -8.49 – -7.07, *p* < 0.001). This profound decrease highlights the effectiveness of the surgical interventions undertaken.

Regarding the type of surgical intervention, individuals undergoing PLIF exhibited a lower baseline pain severity, exceeding that of ALIF patients by 0.93 points *(CI 95*%: -1.59 – -0.27, *p* = 0.006). Notably, the interaction analysis between surgical type and time PLIF revealed an additional pain reduction of 1.41 points at three months post-operation (*CI 95%*: 0.53 – 2.29, *p* = 0.002). This suggests that PLIF, despite a lower initial pain score, may afford less longitudinal pain relief compared to ALIF.

## 2.3.4 . NRS for numbness as the outcome

The robust linear regression analysis examining NRS scores for numbness as an outcome provides valuable insights into the factors influencing postoperative numbness.

The intercept, representing the baseline numbness score for reference levels of the predictors, was significantly high at 4.15 (*CI 95*%: 3.23 – 5.08, *p* < 0.001). This suggests that patients typically experienced moderate numbness before any treatment.

A significant reduction in numbness scores was observed three months after surgery, with a decrease of 3.77 points (*CI 95*%: -4.85 – -2.70, *p* < 0.001). This underscores the efficacy of surgical intervention in alleviating numbness symptoms.

Sex showed no significant effect on numbness scores, with males reporting a slight increase in numbness over females, but this was not statistically significant (*B* = 0.41, CI 95%: -0.29 – 1.12, *p* = 0.249).

Age slightly reduced numbness, but this was not statistically significant (*B* = -0.02, *CI 95%*: -0.05 – 0.01, *p* = 0.139), suggesting that age may not play a critical role in the perception or resolution of numbness post-surgery.

BMI showed a trend towards increasing numbness, with an increase of 0.06 points, but this did not reach statistical significance (*CI 95%*: -0.01 – 0.13, *p* = 0.096). This indicates a possible but not definitive relationship between higher BMI and increased numbness.

Smoking status showed a tendency towards less numbness in smokers compared to non-smokers, but this was not statistically significant (*B* = -0.50, *CI 95%*: -1.13 – 0.14, *p* = 0.124).

Engagement in physical work showed a slight increase in numbness scores, although again, this was not statistically significant (*B* = 0.39, *CI 95*%: -0.27 – 1.06, *p* = 0.245).

A significant finding was the impact of surgery type. Patients undergoing PLIF reported significantly higher numbness scores compared to those undergoing ALIF at baseline, B = 1.98 points (*CI 95*%: 0.98 – 2.97, *p* < 0.001). However, the interaction term revealed that this effect decreased over time post-surgery, with an additional reduction of 1.45 points in numbness scores at three months for those undergoing PLIF (*CI 95*%: -2.78 – -0.12, *p* = 0.032). This suggests that while PLIF initially results in higher numbness, the surgical approach eventually leads to a significant reduction in numbness over time.

## 2.3.5. NRS for spinal stiffness as the outcome

The robust linear regression analysis addressing the NRS scores for spinal stiffness after surgical intervention reveals several significant predictors affecting the postoperative outcomes.

The intercept of 0.90, (*CI 95%: -*0.26 – 2.06, *p* = 0.129), indicates that the baseline level of spinal stiffness before accounting for other variables is not significantly different from zero at conventional levels.

The time effect at three months after surgery shows a decrease in spinal stiffness by 0.56 units, but this change is not statistically significant (*CI 95%:* -1.91 to 0.79, *p* = 0.414). This implies that, on average, the surgery does not significantly alter spinal stiffness scores at three months post-operation compared to the baseline.

The effect of sex, comparing males to the female reference level, shows a slight increase in stiffness for males by 0.29 units; however, this difference is also not statistically significant (*CI 95*%: -0.59 to 1.18, *p* = 0.517).

Age, centered by the median of 43.0 years, shows a small but statistically significant increase in stiffness scores by 0.05 units for each additional year (*CI 95*%: 0.02 to 0.09, *p* = 0.002). This suggests that older patients tend to experience slightly higher levels of spinal stiffness.

For BMI, centered by the median of 27.0 kg/m^2^, there is a statistically significant decrease in stiffness scores by 0.10 units for each unit increase in BMI (*CI 95%:* -0.19 to -0.01, *p* = 0.027). This indicates that higher BMI is associated with lower baseline spinal stiffness.

Smoking status shows that smokers experience significantly higher spinal stiffness than non-smokers by 1.53 units (*CI 95*%: 0.73 to 2.33, *p* < 0.001), highlighting a robust adverse effect of smoking on spinal stiffness.

Physical activity does not significantly affect spinal stiffness, with a change of 0.07 units (*CI 95*%: -0.76 to 0.91, *p* = 0.865).

The type of surgery also plays a significant role, with patients undergoing PLIF reported significantly higher stiffness at baseline by 1.55 units compared to ALIF (*CI 95*%: 0.30 to 2.79, *p* = 0.015). However, the interaction effect of time and surgery type PLIF at three months after surgery shows a decrease in stiffness by 0.79 units, though this interaction is not statistically significant (CI 95%: -2.46 to 0.87, *p* = 0.349), suggesting that the type of surgical intervention may not significantly impact the long-term outcome in spinal stiffness pain reduction.

## 2.4 Evaluating the effects of demographic factors, smoking, work type, surgery type, and pain intensity on quality of life on a Cantril scale in discopathy patients

The robust linear regression analysis, as presented in the table b.7 in Appendices section, evaluates the influence of various factors on the quality of life measured on the Cantril scale for patients with discopathy.

The intercept of 7.44 (*CI 95*%: 6.43 – 8.45, *p* < 0.001) indicates a relatively high baseline quality of life score before considering the impact of other variables. This suggests that, in general, the participants start with a favorable perception of their quality of life.

The time since surgery shows differentiated effects. There is no significant change in the Cantril score three months after surgery (*B* = 0.25, *CI 95*%: -0.16 – 0.67, *p* = 0.236), indicating that short-term post-surgical outcomes may not significantly affect perceived quality of life. However, the projection into the future shows a significant improvement (*B* = 0.68, *CI 95%*: 0.27 – 1.10, *p* = 0.001), suggesting optimism or anticipated better outcomes over the longer term.

Regarding demographics, the regression coefficient for age indicates a slight but significant decline in quality of life with increasing age (*B* = -0.01, *CI 95*%: -0.02 – -0.00, *p* = 0.003). Sex and BMI do not show significant effects on the Cantril score, indicating that these factors do not differentiate quality of life in this analysis.

Smoking status shows no significant impact (*B* = 0.14, *CI 95*%: -0.07 – 0.34, *p* = 0.188), whereas physical activity appears to enhance quality of life significantly (*B* = 0.33, *CI 95*%: 0.10 – 0.57, *p* = 0.006). This underscores the potential benefits of maintaining physical activity post-surgery.

Different types of surgery show significant differences in outcomes. Patients undergoing PLIF report a lower quality of life at baseline compared to those undergoing ALIF (*B* = -0.66, CI 95%: -1.04 – -0.28, p = 0.001), without significant differences in Cantril score over time.

Other medical conditions also show significant effects. Movement impairments (*B* = -0.43, *CI 95*%: -0.83 – -0.03, *p* = 0.036) and spine pain (*B* = -0.64, *CI 95*%: -0.97 – -0.31, *p* < 0.001) are associated with decreased quality of life. However, numbness in limbs also reduces quality of life but to a lesser extent (*B* = -0.31, *CI 95*%: -0.56 – -0.05, *p* = 0.018).

The VAS score at baseline, reflecting baseline pain intensity, has a positive ASOCIATION with quality of life (*B* = 0.20, *CI 95*%: 0.10 – 0.31, *p* < 0.001). This might indicate that patients with higher pain scores possibly receive more intensive treatment or management, which could improve their perceived quality of life.

# 3. Bibliography

Lenth R (2023). emmeans: Estimated Marginal Means, aka Least-Squares Means. *R package* version 1.8.9, <https://CRAN.R-project.org/package=emmeans>.

Lüdecke D (2023). sjPlot: Data Visualization for Statistics in Social Science. *R package* version 2.8.15, <https://CRAN.R-project.org/package=sjPlot>.

Lüdecke D, Ben-Shachar M, Patil I, Makowski D (2020). “Extracting, Computing and Exploring the Parameters of Statistical Models using R.” *Journal of Open Source Software*, 5(53), 2445. doi:10.21105/joss.02445 <https://doi.org/10.21105/joss.02445>.

Lüdecke D, Ben-Shachar M, Patil I, Waggoner P, Makowski D (2021). “performance: An R Package for Assessment, Comparison and Testing of Statistical Models.” *Journal of Open Source Software*, 6(60), 3139. doi:10.21105/joss.03139

Makowski D, Lüdecke D, Patil I, Thériault R, Ben-Shachar M, Wiernik B (2023). “Automated Results Reporting as a Practical Tool to Improve Reproducibility and Methodological Best Practices Adoption.” *CRAN*. <https://easystats.github.io/report/>.

Patil I (2021). “Visualizations with statistical details: The 'ggstatsplot' approach.” *Journal of Open Source Software*, 6(61), 3167. doi:10.21105/joss.03167

R Core Team (2023). R: A Language and Environment for Statistical Computing. R Foundation for Statistical Computing, Vienna, Austria. <https://www.R-project.org/>.

Sjoberg D, Whiting K, Curry M, Lavery J, Larmarange J (2021). “Reproducible Summary Tables with the gtsummary Package.” *The R Journal*, 13, 570-580. doi:10.32614/RJ-2021-053 <https://doi.org/10.32614/RJ-2021-053>, <https://doi.org/10.32614/RJ-2021-053>.

Venables WN, Ripley BD (2002). Modern Applied Statistics with S, Fourth edition. *Springer, New York*. ISBN 0-387-95457-0, <https://www.stats.ox.ac.uk/pub/MASS4/>.

Wickham H, Bryan J (2023). readxl: Read Excel Files. *R package* version 1.4.3, <https://CRAN.R-project.org/package=readxl>.

Wickham H, François R, Henry L, Müller K, Vaughan D (2023). dplyr: A Grammar of Data Manipulation. *R package* version 1.1.3, <https://CRAN.R-project.org/package=dplyr>.

# Appendices

## Appendix A. Dynamics of pain levels and severity of neurological symptoms of discopathy over time

**Figure A.1.** Temporal dynamics of VAS scores in discopathy patients

**Figure A.2.** Temporal dynamics of NRS scores for sciatica symptoms in discopathy patients

**Figure A.3.** Temporal dynamics of NRS scores for spinal pain symptoms in discopathy patients

**Figure A.4.** Temporal dynamics of NRS scores for limb pain symptoms in discopathy patients

**Figure A.5.** Temporal dynamics of NRS scores for numbness in discopathy patients

**Figure A.6.** Temporal dynamics of NRS scores for spinal stiffness symptoms in discopathy patients

**Figure A.7.** Temporal dynamics of NRS scores for weakened sensation of the limbs in discopathy patients

**Figure A.8.** Temporal dynamics of NRS motor impairments in discopathy patients

**Figure A.9.** Temporal dynamics of NRS drop foot symptoms in discopathy patients

**Figure A.10.** Temporal dynamics of the Cantil score of life satisfaction in patients with discopathy.

## Appendix B. Results of the multivariate regression analysis

## B.1. VAS score as the outcome

**Table B.1.** Robust linear regression coefficients with VAS score as the outcome, *N_obs_* = 224.

| *Predictors* | VAS score | | |
| --- | --- | --- | --- |
|  | *B* | *CI 95%* | *p* |
| (Intercept) | 8.07 | 7.56 – 8.58 | **< 0.001** |
| time [baseline] | Reference level | | |
| time [3 months after surgery] | -6.07 | -6.67 – -5.48 | **< 0.001** |
| sex [female] | Reference level | | |
| sex [male] | -0.15 | -0.54 – 0.23 | 0.434 |
| age,  centered by the median *Mdn* = 43.0 yrs. | 0.01 | -0.00 – 0.03 | 0.103 |
| BMI  centered by the median *Mdn* = 27.0 kg/m^2^ | 0.01 | -0.03 – 0.05 | 0.635 |
| smoking [no] | Reference level | | |
| smoking [yes] | 0.18 | -0.17 – 0.53 | 0.313 |
| physical work [no] | Reference level | | |
| physical work [yes] | 0.03 | -0.34 – 0.39 | 0.882 |
| surgery type [ALIF] | Reference level | | |
| surgery type [PLIF] | -0.11 | -0.66 – 0.44 | 0.690 |
| time [3 months after surgery] ×  surgery type [PLIF] | 1.66 | 0.93 – 2.40 | **< 0.001** |

*Note:* *B* – regression coefficient (non-standardized); *CI 95%* – confidence interval 95%; *p* – p-value of statistical test, *N_obs_* – number of observation (with repeated measures)

## B.2. NRS score for sciatica as the outcome

**Table B.2.** Robust linear regression coefficients with NRS sciatica score as the outcome, *N_obs_* = 224.

| *Predictors* | NRS sciatica | | |
| --- | --- | --- | --- |
|  | *B* | *CI 95%* | *p* |
| (Intercept) | 7.52 | 6.86 – 8.19 | **< 0.001** |
| sex [female] | Reference level | | |
| sex [male] | 0.02 | -0.49 – 0.53 | 0.944 |
| age  centered by the median *Mdn* = 43.0 yrs. | -0.02 | -0.04 – -0.00 | **0.019** |
| BMI  centered by the median *Mdn* = 27.0 kg/m^2^ | 0.03 | -0.03 – 0.08 | 0.310 |
| smoking [no] | Reference level | | |
| smoking [yes] | 0.33 | -0.13 – 0.79 | 0.154 |
| physical work [no] | Reference level | | |
| physical [yes] | -0.10 | -0.58 – 0.38 | 0.684 |
| time [baseline] | Reference level | | |
| time [3 months after surgery] | -7.13 | -7.90 – -6.36 | **< 0.001** |
| surgery type [ALIF] | Reference level | | |
| surgery type [PLIF] | -0.09 | -0.81 – 0.62 | 0.795 |
| time [3 months after surgery] × surgery type [PLIF] | 0.59 | -0.36 – 1.55 | 0.222 |

*Note:* *B* – regression coefficient (non-standardized); *CI 95%* – confidence interval 95%; *p* – p-value of statistical test, *N_obs_* – number of observation (with repeated measures)

## B.3. NRS for spinal pain as the outcome

**Table B.3.** Robust linear regression coefficients with NRS spinal pain score as the outcome, *N_obs_* = 224.

| *Predictors* | NRS spinal pain | | |
| --- | --- | --- | --- |
|  | *B* | *CI 95%* | *p* |
| (Intercept) | 8.29 | 7.66 – 8.92 | **< 0.001** |
| sex [female] | Reference level | | |
| sex [male] | -0.03 | -0.51 – 0.45 | 0.910 |
| age  centered by the median *Mdn* = 43.0 yrs. | 0.02 | 0.00 – 0.04 | **0.015** |
| BMI  centered by the median *Mdn* = 27.0 kg/m^2^ | 0.01 | -0.04 – 0.06 | 0.700 |
| smoking [no] | Reference level | | |
| smoking [yes] | 0.33 | -0.10 – 0.77 | 0.133 |
| physical work [no] | Reference level | | |
| physical [yes] | -0.32 | -0.78 – 0.13 | 0.160 |
| time [baseline] | Reference level | | |
| time [3 months after surgery] | -5.18 | -5.92 – -4.45 | **< 0.001** |
| surgery type [ALIF] | Reference level | | |
| surgery type [PLIF] | -0.59 | -1.27 – 0.09 | 0.090 |
| time [3 months after surgery] × surgery type [PLIF] | 1.44 | 0.53 – 2.34 | **0.002** |

*Note:* *B* – regression coefficient (non-standardized); *CI 95%* – confidence interval 95%; *p* – p-value of statistical test, *N_obs_* – number of observation (with repeated measures)

## B.4. NRS for limb pain as the outcome

**Table B.4.** Robust linear regression coefficients with NRS limb pain score as the outcome, *N_obs_* = 224.

| *Predictors* | NRS limb pain | | |
| --- | --- | --- | --- |
|  | *B* | *CI 95%* | *p* |
| (Intercept) | 7.72 | 7.11 – 8.33 | **< 0.001** |
| sex [female] | Reference level | | |
| sex [male] | 0.11 | -0.36 – 0.58 | 0.639 |
| age  centered by the median *Mdn* = 43.0 yrs. | 0.02 | 0.00 – 0.04 | **0.032** |
| BMI  centered by the median *Mdn* = 27.0 kg/m^2^ | 0.01 | -0.04 – 0.05 | 0.795 |
| smoking [no] | Reference level | | |
| smoking [yes] | 0.40 | -0.02 – 0.82 | 0.065 |
| physical work [no] | Reference level | | |
| physical [yes] | 0.43 | -0.01 – 0.87 | 0.054 |
| time [baseline] | Reference level | | |
| time [3 months after surgery] | -7.78 | -8.49 – -7.07 | **< 0.001** |
| surgery type [ALIF] | Reference level | | |
| surgery type [PLIF] | -0.93 | -1.59 – -0.27 | **0.006** |
| time [3 months after surgery] ×  surgery type [PLIF] | 1.41 | 0.53 – 2.29 | **0.002** |

*Note:* *B* – regression coefficient (non-standardized); *CI 95%* – confidence interval 95%; *p* – p-value of statistical test, *N_obs_* – number of observation (with repeated measures)

## B.5. NRS for numbness as the outcome

**Table B.5.** Robust linear regression coefficients with NRS numbness score as the outcome, *N_obs_* = 224.

| *Predictors* | NRS numbness | | |
| --- | --- | --- | --- |
|  | *B* | *CI 95%* | *p* |
| (Intercept) | 4.15 | 3.23 – 5.08 | **< 0.001** |
| time [baseline] | **Reference level** | | |
| time [3 months after surgery] | -3.77 | -4.85 – -2.70 | **< 0.001** |
| sex [female] | **Reference level** | | |
| sex [male] | 0.41 | -0.29 – 1.12 | 0.249 |
| age  centered by the median *Mdn* = 43.0 yrs. | -0.02 | -0.05 – 0.01 | 0.139 |
| BMI  centered by the median *Mdn* = 27.0 kg/m^2^ | 0.06 | -0.01 – 0.13 | 0.096 |
| smoking [no] | **Reference level** | | |
| smoking [yes] | -0.50 | -1.13 – 0.14 | 0.124 |
| physical [no] | **Reference level** | | |
| physical [yes] | 0.39 | -0.27 – 1.06 | 0.245 |
| surgery type [ALIF] | **Reference level** | | |
| surgery type [PLIF] | 1.98 | 0.98 – 2.97 | **<0.001** |
| time [3 months after surgery] ×  surgery type [PLIF] | -1.45 | -2.78 – -0.12 | **0.032** |

*Note:* *B* – regression coefficient (non-standardized); *CI 95%* – confidence interval 95%; *p* – p-value of statistical test, *N_obs_* – number of observation (with repeated measures)

## B.6. NRS for spinal stiffness as the outcome

**Table B.6.** Robust linear regression coefficients with NRS spinal stiffness score as the outcome, *N_obs_* = 224.

| *Predictors* | NRS spinal stiffness | | |
| --- | --- | --- | --- |
|  | *B* | *CI 95%* | *p* |
| (Intercept) | 0.90 | -0.26 – 2.06 | 0.129 |
| time [baseline] | **Reference level** | | |
| time [3 months after surgery] | -0.56 | -1.91 – 0.79 | 0.414 |
| sex [female] | **Reference level** | | |
| sex [male] | 0.29 | -0.59 – 1.18 | 0.517 |
| age  centered by the median *Mdn* = 43.0 yrs. | 0.05 | 0.02 – 0.09 | **0.002** |
| BMI  centered by the median *Mdn* = 27.0 kg/m^2^ | -0.10 | -0.19 – -0.01 | **0.027** |
| smoking [no] | **Reference level** | | |
| smoking [yes] | 1.53 | 0.73 – 2.33 | **<0.001** |
| physical [no] | **Reference level** | | |
| physical [yes] | 0.07 | -0.76 – 0.91 | 0.865 |
| surgery type [ALIF] | **Reference level** | | |
| surgery type [PLIF] | 1.55 | 0.30 – 2.79 | **0.015** |
| time [3 months after surgery] ×  surgery type [PLIF] | -0.79 | -2.46 – 0.87 | 0.349 |

*Note:* *B* – regression coefficient (non-standardized); *CI 95%* – confidence interval 95%; *p* – p-value of statistical test, *N_obs_* – number of observation (with repeated measures)

## B.7. Cantril score as the outcome

**Table B.7.** Robust linear regression coefficients with Cantril score as the outcome, *N_obs_* = 336.

| *Predictors* | Cantril score | | |
| --- | --- | --- | --- |
|  | *B* | *CI 95%* | *p* |
| (Intercept) | 7.44 | 6.43 – 8.45 | **<0.001** |
| time [baseline] | **Reference level** | | |
| time [3 months after surgery] | 0.25 | -0.16 – 0.67 | 0.236 |
| time [in future] | 0.68 | 0.27 – 1.10 | **0.001** |
| sex [female] | **Reference level** | | |
| sex [male] | 0.06 | -0.17 – 0.29 | 0.613 |
| age  centered by the median *Mdn* = 43.0 yrs. | -0.01 | -0.02 – -0.00 | **0.003** |
| BMI  centered by the median *Mdn* = 27.0 kg/m^2^ | 0.01 | -0.01 – 0.04 | 0.277 |
| smoking [no] | **Reference level** | | |
| smoking [yes] | 0.14 | -0.07 – 0.34 | 0.188 |
| physical [no] | **Reference level** | | |
| physical [yes] | 0.33 | 0.10 – 0.57 | **0.006** |
| surgery type [ALIF] | **Reference level** | | |
| surgery type [PLIF] | -0.66 | -1.04 – -0.28 | **0.001** |
| movement impairments [no] | **Reference level** | | |
| movement impairments [yes] | -0.43 | -0.83 – -0.03 | **0.036** |
| sensory impairments [no] | **Reference level** | | |
| sensory impairments [yes] | 0.16 | -0.20 – 0.53 | 0.374 |
| numbness in limbs [no] | **Reference level** | | |
| numbness in limbs [yes] | -0.31 | -0.56 – -0.05 | **0.018** |
| spine pain [no] | **Reference level** | | |
| spine pain [yes] | -0.64 | -0.97 – -0.31 | **<0.001** |
| lower limb pain [no] | **Reference level** | | |
| lower limb pain [yes] | 0.15 | -0.11 – 0.40 | 0.263 |
| sphincter dysfunctions [no] | **Reference level** | | |
| sphincter dysfunctions [yes] | -0.19 | -0.77 – 0.39 | 0.524 |
| VAS score at baseline | 0.20 | 0.10 – 0.31 | **< 0.001** |
| time [in future] × surgery type [PLIF] | 0.37 | -0.15 – 0.88 | 0.163 |
| time [3 months after surgery] ×  surgery type [PLIF] | 0.39 | -0.13 – 0.91 | 0.137 |

*Note:* *B* – regression coefficient (non-standardized); *CI 95%* – confidence interval 95%; *p* – p-value of statistical test, *N_obs_* – number of observation (with repeated measures)
